# The *C. elegans* Male Exercises Directional Control during Mating through Cholinergic Regulation of Sex-Shared Command Interneurons

**DOI:** 10.1371/journal.pone.0060597

**Published:** 2013-04-05

**Authors:** Amrita L. Sherlekar, Abbey Janssen, Meagan S. Siehr, Pamela K. Koo, Laura Caflisch, May Boggess, Robyn Lints

**Affiliations:** 1 Department of Biology, Texas A & M University, College Station, Texas, United States of America; 2 School of Mathematical and Statistical Sciences,Arizona State University, Tempe, Arizona, United States of America; Brown University/Harvard, United States of America

## Abstract

**Background:**

Mating behaviors in simple invertebrate model organisms represent tractable paradigms for understanding the neural bases of sex-specific behaviors, decision-making and sensorimotor integration. However, there are few examples where such neural circuits have been defined at high resolution or interrogated.

**Methodology/Principal Findings:**

Here we exploit the simplicity of the nematode *Caenorhabditis elegans* to define the neural circuits underlying the male’s decision to initiate mating in response to contact with a mate. Mate contact is sensed by male-specific sensilla of the tail, the rays, which subsequently induce and guide a contact-based search of the hermaphrodite’s surface for the vulva (the vulva search). Atypically, search locomotion has a backward directional bias so its implementation requires overcoming an intrinsic bias for forward movement, set by activity of the sex-shared locomotory system. Using optogenetics, cell-specific ablation- and mutant behavioral analyses, we show that the male makes this shift by manipulating the activity of command cells within this sex-shared locomotory system. The rays control the command interneurons through the male-specific, decision-making interneuron PVY and its auxiliary cell PVX. Unlike many sex-shared pathways, PVY/PVX regulate the command cells via cholinergic, rather than glutamatergic transmission, a feature that likely contributes to response specificity and coordinates directional movement with other cholinergic-dependent motor behaviors of the mating sequence. PVY/PVX preferentially activate the backward, and not forward, command cells because of a bias in synaptic inputs and the distribution of key cholinergic receptors (encoded by the genes *acr-18, acr-16* and *unc-29*) in favor of the backward command cells.

**Conclusion/Significance:**

Our interrogation of male neural circuits reveals that a sex-specific response to the opposite sex is conferred by a male-specific pathway that renders subordinate, sex-shared motor programs responsive to mate cues. Circuit modifications of these types may make prominent contributions to natural variations in behavior that ultimately bring about speciation.

## Introduction

Courtship and mating are among the most elaborate of behaviors displayed in the animal kingdom [1]. The ultimate goal of the male in these behaviors is to fertilize oocytes. However, achieving this goal often requires the execution of a behavioral sequence, progression through which depends on detection of appropriate conspecific cues and the expression of appropriate behavioral responses [2]. These sequences serve to ensure that copulation occurs between conspecifics and that unworthy or inappropriate partners are rejected. As such, the study of mating behavior can provide valuable insight into understanding not only the neural bases of sex-specific behaviors, but also mechanisms underlying decision-making, sensorimotor integration and the coordinated timing of sequenced motor behaviors. In many species, delineation of the underlying circuitry and its functional interrogation are hampered, however, by nervous system complexity and relative genetic intractability.

The nematode *C. elegans* is an attractive model for exploring the circuits controlling sex-specific behaviors. The two sexes, male and hermaphrodite, have comparatively simple nervous systems, consisting of only 302 and 383 neurons, respectively 294 of which are sex-shared (a.k.a. core neurons) [3]–[5]. The connectivity of the hermaphrodite nervous system has been described with single cell resolution [6], [7] and, recently, an equally detailed map of the male posterior nervous system has been completed [3]. These wiring diagrams, combined with the amenability of *C. elegans* to genetic and optogenetic manipulation, enables the functional dissection of circuits supporting specific behaviors, with single cell resolution and in the context of a freely behaving animal (reviewed in Xu and Kim, 2011 [8]).

**Figure 1:**
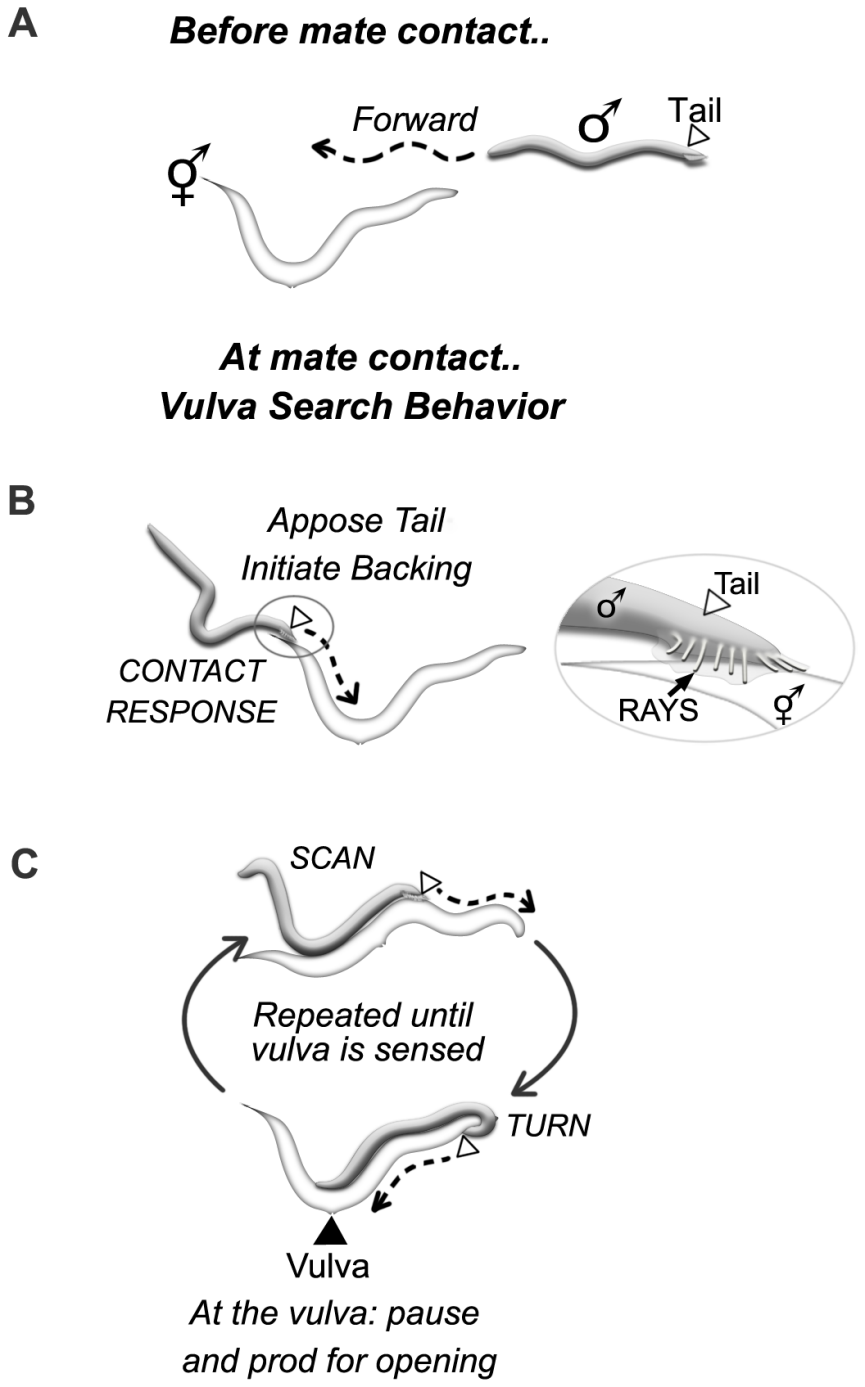
Male mating behavior is characterized by distinct patterns of locomotion. A cartoon depicting the key changes observed in male movement and body posture that are triggered by mate contact. ***A.*** In the absence of mate contact, male locomotion resembles that of the hermaphrodite: the male moves with a forward locomotion bias and the sinusoidal body wave driving movement propagates along the full length of the body. ***B.*** Contact with a mate via the male tail induces contact response: the male presses his tail against the hermaphrodite surface and commences backward movement. ***C.*** Backward locomotion continues until the vulva is sensed, whereupon the male pauses and prods for the vulva slit opening with his copulatory spicules. The male sensory rays (shown in the inset for *B*), which sense hermaphrodite contact, are essential for the induction and maintenance of tail apposition and for directional control on the hermaphrodite surface.

The *C. elegans* hermaphrodite is essentially a female that can make a limited supply of sperm for self-fertilization. The hermaphrodite can also fertilize her oocytes using sperm acquired through copulation with a male [9]. The *C. elegans* male mates the hermaphrodite using a goal-oriented behavioral sequence that is composed of sex-specific motor behaviors [10], [11]. In this study, we explore the circuitry controlling the male’s decision to initiate mating in response to physical contact with a mate. Prior to a mate encounter, the locomotory patterns of the male are similar to that of the hermaphrodite. He moves through his environment with a predominantly forward directional bias and his movement is powered by propagation of a dorsal-ventral sinusoidal wave along the body (Fig. 1A). If mate contact prompts the mating choice, the male initiates a contact-based search of the hermaphrodite’s surface for the vulva region, the vulva search. External sensilla on the male tail, the male sensory rays, are responsible for inducing vulva search behavior and guiding its trajectory in response to hermaphrodite surface cues (Fig. 1B–1C) [12]. Ray stimulation causes the male to abruptly cease forward movement and reverse onto the hermaphrodite, apposing his tail against her surface (Fig. 1B). This action brings the rays and the vulva-sensing sensilla of the tail (the hook and the post-cloacal sensilla – p.c.s.) into direct contact with the hermaphrodite cuticle. With his tail apposed, the male moves systematically over the hermaphrodite’s surface with a backward directional bias so that his sensilla-laden tail leads the way (Fig. 1C). Both this extended bout of backward movement and tail apposition posture are male-specific motor behaviors. Their induction also sex-specifically alters propagation of the sinusoidal wave, such that it does not progress to the tail region and may be variably absent from the posterior half of the body during mating. The vulva search continues until the hook and p.c.s. sense the vulva area and induce the male to pause there and begin to prod for the vulva slit with his copulatory spicules [11], [13], [14].

The hermaphrodite locomotory system has been extensively studied and homologous cellular components are present in the male nervous system, raising the possibility that male movement during mating may be supported by this sex-shared circuitry [4], [5], [7]. However, the male nervous system also contains 89 additional neurons that are male-specific, mainly distributed through the tail and the ventral nerve cord [3]–[5]. The extent to which these male-specific cells contribute to locomotory control during mating is the question of current interest. In this study, we show that the rays regulate directional movement during the vulva search using a male-specific pathway that converges on the sex-shared locomotory system, rendering it responsive to mate-derived sensory signals. Our data suggest that ray activation triggers the forward-to-backward directional switch by preferentially upregulating the activity of backward command interneurons in this system. The rays act through male-specific decision-making interneuron PVY and its auxiliary cell PVX. These interneurons define the convergence point for multiple sensory pathways, both sex-shared and male-specific, suggesting that PVY and PVX may be a pivotal site for integrating and prioritizing various sensory cues to generate the appropriate behavioral response.

## Results

### Backward Locomotion during Mating Depends on Sex-shared Command Interneurons

**Figure 2:**
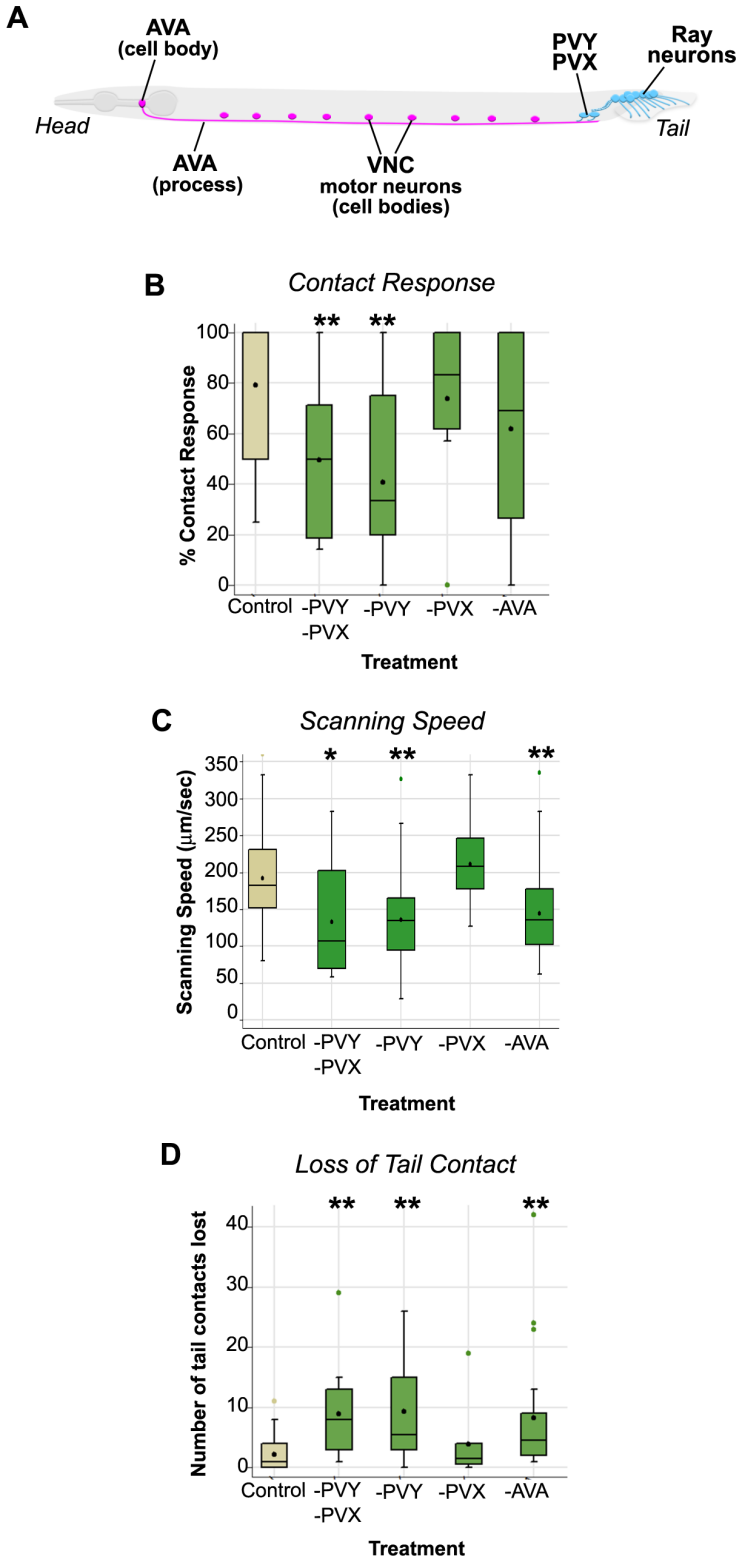
Male movement during mating depends on sex-shared and male-specific interneurons. ***A.*** Schematic of an adult male (lateral view, left side) showing the anatomical location of cells ablated in experiments shown in *B–D* and in Fig. 3. Sex-shared cells (pink); male-specific cells (blue). The backward command interneurons (only the left AVA neuron is shown) have cell bodies in the head and send a process along the ventral nerve cord (VNC) where they synapse with motor neurons required for locomotion (the distribution of cell bodies for only a single motor neuron class is shown). PVY and PVX (located in the male pre-anal ganglion) receive inputs from the ray sensory neurons and have outputs onto the command interneuron processes. ***B–D.*** The impact of cell-specific ablations on three aspects of vulva search behavior related to locomotory control. ***B*** **.** Contact response efficiency. ***C.*** Scanning speed. ***D.*** The number of times tail contact was lost per mating. The X-axis indicates the cells ablated in each treatment. A box plot representation of the data is shown, with median and mean values indicated by the line and the black dot within the box, respectively. Comparisons to the control (mock-ablated) were made using a ranksum test. The number of males assayed for each treatment, n: Control = 63; -PVY-PVX = 15; -PVY = 19; -PVX = 8; -AVA = 22. Significance, *p<0.05; **p<0.005.

In the *C. elegans* hermaphrodite considerable progress has been made in understanding the cellular and molecular bases for directional control of movement. Laser ablation experiments have uncovered a small group of interneurons that play a prominent role in the choice between backward and forward locomotion [15]–[18]. These interneurons, termed command interneurons, can be divided into two functional classes. The backward command interneurons, AVA, AVD and AVE left/right (L/R) bilateral pairs, promote backward locomotion. The forward command interneurons, AVB and PVC L/R bilateral pairs, promote forward movement. The forward and backward command interneurons confer movement directionality by stimulating distinct pools of ventral nerve cord motor neurons (Fig. 2A), dedicated to forward and backward movement, respectively [15], [19]–[21]. Motor neuron activity, in turn, regulates the sex-shared body wall muscles to produce the sinusoidal wave. The command interneurons are the postsynaptic targets of a number of sensory pathways that promote movement either towards or away from specific stimuli by preferentially stimulating forward or backward command cells, respectively [7], [22]–[25]. During spontaneous locomotion (locomotion in the absence of specific stimuli), the worm moves with a forward directional bias but occasionally moves backward (i.e. makes reversals) to change trajectory [26]. It is proposed that this forward locomotion bias is produced by an intrinsic imbalance of ventral nerve cord motor neuron activities favoring the forward motor pathway. This imbalance is established in part by gap junction connections [21] and in part by descending inputs from a second directional control pathway, the disinhibitory pathway [25].

The command interneurons and motor components of the locomotory system are also present in the male nervous system [3]–[5]. In the pre-anal ganglion (PAG) of the male tail, the command cell processes receive direct inputs from a number of male-specific interneurons that are postsynaptic targets of the ray neurons (Fig. 2A). Although these male inputs target both backward and forward command interneurons, these connections are heavily weighted in favor of the backward command interneurons, in particular AVA. This suggests that the rays may promote reversal by preferentially activating AVA. To test this, we eliminated the AVA interneurons in males using laser-mediated cell-specific ablation then examined its impact on male mating behavior (Materials and Methods). AVA interneurons are born in the embryo, however, presynaptic male-specific neurons (born in the third larval stage (L3)) do not connect with AVA until the stage prior to adulthood and sexual maturation, L4. Therefore, we performed these AVA ablations on L4 males. Mock-ablated (Control) and AVA-ablated L4 males were allowed to mature into adults overnight in the absence of hermaphrodites, so they were virgin, 1-day old adults at the time of assay. All male populations examined in this study were isolated in this way to ensure that they were sexually inexperienced and of the same age as, anecdotally, we have observed that these factors significantly affect the probability of vulva search initiation. Mating behavior assays were performed as follows: a single mock- or cell-ablated virgin adult male was placed on a bacterial mating lawn with five virgin, 1-day old adult hermaphrodites. Genetically wild type hermaphrodites often dart or move during the male’s mating attempts [27], making quantification of male locomotory behavior difficult. To reduce hermaphrodite movement during mating, we used *unc-64* hermaphrodites as mating partners, which are sluggish due to a mutation in the *C. elegans* syntaxin gene [28]. Male behavior during mating was digitally recorded for 15 mins or until ejaculation occurred, which ever happened first [12]. We paid particular attention to three aspects of vulva search behavior that would indicate the efficiency of forward to backward switching: the frequency with which backward movement was induced by initial contact (a.k.a. contact response; Fig. 2B) and the strength and duration of the backward movement during the search (scanning speed and sustained tail contact, respectively; Fig. 2C, 2D).

Mock-ablated males typically responded on the first or second contact by placing their tail, ventral side down, against the hermaphrodite and commencing backing (Video S1). Consequently these control males had a median contact response success rate of 100% (i.e. 1 out of 1 contacts resulted in a complete contact response, namely both tail apposition and initiation of backward movement; Fig. 2B). Using these motor behaviors, control males then moved along the hermaphrodite’s anterior-posterior axis, a behavior referred to as scanning (Fig. 1C). If males reached the hermaphrodite head or tail without sensing the vulva, they made a sharp turn, without losing tail contact, and scanned along the other side of the hermaphrodite, again moving with backward locomotion but in the opposite direction. We calculated the scanning speed of control males (in µm/sec) by dividing the length of the hermaphrodite by the time required for a male to scan the length (see Materials and Methods for details). As encountering the vulva can induce pausing (and consequently affect scanning time), scanning speed measurements were obtained from only the non-vulva side. Control males scanned a hermaphrodite length at a median speed of 180 µm/sec (Fig. 2C). Control males typically maintained tail contact with the hermaphrodite for the entire search (Fig. 2D; median number of lost contacts during scanning = 1).

Ablation of AVA caused locomotion defects in males that were apparent even when males were not engaged in mating. These defects resembled those reported for AVA-ablated hermaphrodites [15]. Specifically, during spontaneous locomotion, AVA-ablated males had difficulty switching from forward to backward movement and consequently reversed only a short distance before reverting to forward movement. Backward movement defects were also evident in vulva search behavior. AVA-ablated males exhibited highly variable contact response efficiency (-AVA treatment, Fig. 2B). When scanning was initiated successfully, the scanning speed of AVA-ablated males was significantly slower than that of control males (-AVA treatment: median scanning speed = 140 µm/sec *cf.* control males = 180 µm/sec; Fig. 2C). In addition, AVA-ablated males lost tail contact during scanning at a significantly higher rate than controls, possibly because the uncoordinated phenotype resulting from AVA ablation interferes with tail apposition (-AVA treatment: median number of lost contacts = 5 *cf.* control males = 1; Fig. 2D). These results suggest that the rays depend significantly on the sex-shared AVA command interneurons to induce backward movement during the vulva search. However, our observation that AVA-ablated males can exhibit limited and stochastic backward movement during mating also reveals that the rays may additionally act through other interneurons involved in directional control. The stochastic nature of ablated-male defects suggests that, in some instances, activity of these alternative pathways is sufficient to drive backward movement, whereas at other moments it is not.

### Male-specific Interneuron PVY Promotes Backward Locomotion during Mating

**Figure 3:**
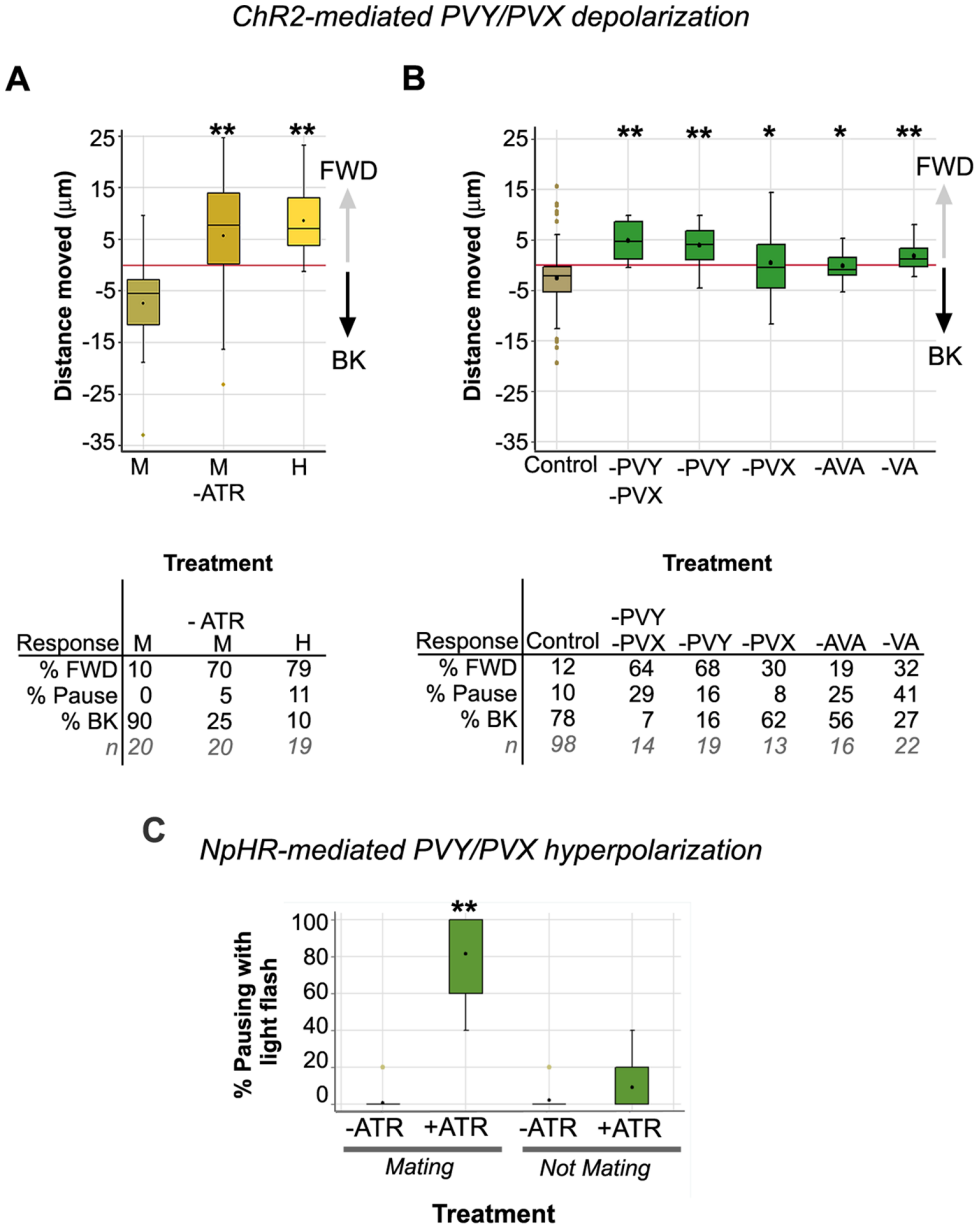
Optogenetic manipulation of PVY and PVX activity affects male movement. ***A, B.*** PVY and PVX artificial stimulation, using ChR2, induces backward locomotion that is male-specific and depends on sex-shared locomotory system cells. The graphs show the impact of ChR2 activation on *Pnlp-14::ChR2-YFP* transgenic animal locomotion. Except for H (hermaphrodites) in *(A)*, all animals tested were transgenic males (in *(A),* male treatments are designated “M”). Except for the “-ATR M” control treatment in *(A)*, all animals were cultured and assayed in the presence of OP50 *E.coli* food supplemented with ATR. The X-axis indicates the food supplementation conditions (+ATR or –ATR), animal sex or which cells were ablated. The controls in *(B)* correspond to mock-ablated animals. The Y-axis shows the distance traveled (in µm) in response to the flash, with the negative values indicating backward (BK) movement and the positive values indicating forward (FWD) movement (see Materials and Methods). Statistical comparisons to the relevant controls were made using a ranksum test for differences in the median. Tabled below each graph is the percentage of animals in each treatment that backed, paused or continued to move forward (see Materials and Methods for the µm range of each category). *n* is the number of worms assayed in each treatment. ***C*** **.** Artificial hyperpolarization of PVY and PVX blocks backing in the context of mating. Shown is the pausing frequency of *Pnlp-14::NpHR-EYFP* males in response to yellow light flashes. The X-axis shows the food supplementation and mating (Mating or Not Mating) conditions used. The Y-axis indicates the percentage of light flashes (out of 5) that induced pausing. *n*: -ATR Mating, 26; +ATR Mating, 28; -ATR Not Mating, 10; +ATR Not Mating, 10. Comparisons between –ATR and +ATR treatments were made using a ranksum test. Significance, *p<0.05; **p<0.005.

We next investigated the neural pathways by which the rays control AVA. In the male PAG, two major ray neuron targets, the male-specific interneurons PVY and PVX, have significant inputs onto command interneuron processes (Fig. 2A; [3]). These inputs are biased in favor of the AVA backward command interneurons such that the ratio of synaptic inputs onto AVA, relative to the AVB forward command interneurons is 2∶1 [3]. To test whether PVY and/or PVX represent the major route by which the rays control the command cells, we ablated one or both male-specific interneurons and assessed the impact on mating behavior. In males where only PVY was ablated, contact response efficiency and scanning speed were significantly reduced compared to control males (-PVY treatment: median % contact response = 30%, Fig. 2B; median scanning speed 135 µm/sec, Fig. 2C; Video S2). PVY-ablated males also lost tail contact at a significantly higher frequency than control males during the search (median number of contacts lost = 5; Fig. 2D). These various locomotion defects and their stochastic expression resembled the behavioral phenotype of AVA-ablated males, consistent with the model that PVY promotes backwards movement by acting through AVA neurons. However, in contrast to AVA-ablated males, PVY-ablated animals had no obvious defects in reversal when not engaged in mating (data not shown). Thus, PVY is only required for locomotory control in response to ray-mediated mate contact. Although PVX also has inputs onto AVA, its ablation had no obvious affect on vulva search behavior. Males lacking PVX had superficially wild type mating behavior (-PVX treatment, Fig. 2B–2D) and males lacking both PVX and PVY (-PVY-PVX treatment, Fig. 2B–2D) were not significantly different from males lacking PVY only. Together, these ablation experiments reveal that PVY promotes backward movement during the vulva search, while PVX may have a functionally redundant or subtle role in the process. Like the AVA-ablated males, males lacking PVY could still reverse along the hermaphrodite, albeit erratically and slowly. Thus, although PVY and AVA are important for backward moment during mating, other ray neuron targets may be able to partially compensate for their absence.

### Optogenetic Manipulation of PVY and PVX Activity Affects Male Movement

To gain further insight into the functional properties of PVY and PVX, we artificially activated these interneurons in solitary males using the heterologous light-inducible cation channel ChannelRhodopsin-2 (ChR2) from *Chlamydomonas reinhardtii* [29]. To target expression of ChR2 to PVY and PVX, we placed a YFP-tagged ChR2 transgene *(ChR2-YFP)* under the control of the *nlp-14* promoter (*Pnlp-14*; [30]). Previous studies have shown that this particular *nlp-14* promoter sequence drives expression of GFP reporters to a subset of nervous system cells common to both sexes: the sensory neurons ASI, ASK, ASE, PHA, two retrovesicular ganglion neurons, ventral nerve cord motor neurons and the interneuron PVT [30]. We find that in the male *Pnlp-14::GFP* reporters are additionally expressed in male-specific PVY, PVX and two male-specific dorsal rectal ganglion cells. However, we observed that the *ChR2-YFP* transgene, when placed under the control of this same *nlp-14* promoter region, is expressed in only a fraction of these cells, being notably absent from the ventral nerve cord motor neurons, PHA and often the male dorsal rectal ganglion neurons. The limited expression of CHR2-YFP, compared to GFP, may be because the former lacks introns and consequently may be less efficiently processed in some cell types. Retinal is an essential co-factor for ChR2 and as *C. elegans* does not endogenously synthesize this compound, it must be provided in their food in the form of all-*trans-*retinal (ATR) in order for the animal to generate functional ChR2 (Materials and Methods). To perform the artificial activation assays, 1-day old adult, virgin *Pnlp-14::ChR2-YFP* males were individually exposed to a 500 msec flash of blue light (470/40 nm wavelength) while moving forward. Since blue light is an aversive stimulus for *C. elegans* and can alone elicit an avoidance response (either forward or backward movement away from the source), these transgenic animals also carried a mutation in the *lite-1* gene (*lite-1(ce314)*), which encodes a putative ultraviolet receptor [31]. Animals carrying *lite-1(ce314)* are substantially less avoidant of blue light. The behavior of transgenic animals was digitally recorded during the assay and the distance they traveled (either forward or backward) from the time of the flash was determined by analyzing video frames (as described in Materials and Methods). When exposed to the light flash, 90% of *Pnlp-14::ChR2-YFP* males immediately reversed and traveled a median distance of -5 µm, with the negative value indicating that they reversed away from their position at the time of the flash (M treatment, Fig. 3A, Video S3). Through a series of control experiments we determined that this reversal response was specifically due to activation of male-specific interneurons PVY and PVX. First, this robust reversal behavior was not a non-specific response to the light flash, as the substantial majority of males expressing non-functional ChR2 did not alter their behavior and continued moving forward (-ATR, M treatment, Fig. 3A, Video S4). Second, *Pnlp-14::ChR2-YFP* transgenic hermaphrodites, which lack PVY and PVX, did not respond to the light flash (H treatment, Fig. 3A). Moreover, *Pnlp-14::ChR2-YFP* males lacking PVY and PVX cells, due to their specific ablation with the laser, did not reverse in response to the light flash and continued moving forward (-PVY-PVX treatment, Fig. 3B). These experiments demonstrate that artificial activation of PVY and PVX is sufficient to overcome the forward directional bias and induce backward movement.

We next used the ChR2 artificial activation system to further explore the relative contribution of PVY and PVX to male reversal behavior. In the mating assays discussed above, eliminating PVY alone significantly disrupted backward locomotion during the vulva search (Fig. 2B–2D). Consistent with this, few *Pnlp-14::ChR2-YFP* males ablated for PVY reversed when exposed to the light flash (16%, -PVY treatment, Fig. 3B), even though PVX was present and presumably activated. While activation of PVX alone had little impact, PVX co-stimulation with PVY enhanced the robustness of the reversal response, as males with only PVY (-PVX) showed greater variability in their response. Thus, while these experiments confirm that PVY is a crucial effecter of backward locomotion, they also reveal a function for PVX as an auxiliary cell, a role that may be readily compensated for by other ray targets in the context of mating if PVX is absent (Fig. 2).

The ChR2 experiments above showed that PVY+PVX activation is sufficient to induce the switch from forward to backward locomotion. We next asked whether PVY and PVX activity were required continuously during the vulva search to drive backward movement. To test this, we used the heterologous light-inducible hyperpolarizing channel NpHR, from *Natronomonas pharaonis* [32], [33], to inhibit PVY and PVX while the male was performing the search. These experiments were performed on *lite-1(ce314)* transgenic males in which NpHR expression was targeted to PVY and PVX by placing an *NpHR-EYFP* transgene under the control of the *nlp-14* promoter. Under the control of this promoter, NpHR-YFP is expressed in the same constellation of cells as ChR2-YFP (described above). Like ChR2, NpHR function depends on the presence of ATR so males grown in the absence of ATR provide a convenient negative control for any non-specific effect of light exposure. In each mating assay, a single virgin 1-day old adult transgenic male was placed with 5 virgin adult *unc-64; lite-1* hermaphrodites on a mating lawn and allowed to initiate mating. We activated NpHR during scanning by exposing the male to a 500 msec flash of yellow light (540/25 nm wavelength) at 5, evenly spaced, time intervals and counted the number of times pausing coincided with a pulse (Fig. 3C). *Pnlp-14::NpHR-EYFP* males, grown in the presence of ATR, paused in response to 4 out of 5 flashes, on average (+ ATR+mating treatment: mean pausing frequency = 80%, Video S5). In contrast, *Pnlp-14::NpHR-EYFP* control males, grown in the absence of ATR, largely ignored the light pulses and continued scanning, pausing at a mean frequency of only 1% (-ATR+mating treatment, Fig. 3C, Video S6). These data therefore argue that PVY and PVX function is required continuously during the vulva search to maintain backward movement. To test whether NpHR activation could induce pausing when males were not engaged in mating, we flashed *Pnlp-14::NpHR-EYFP* males (grown in the presence of ATR) during spontaneous locomotion while they were reversing. These males rarely paused suggesting that the pausing induced during mating was due to hyperpolarization of cells that are normally active only when the rays sense mate contact (+ATR+no mating treatment: mean pausing frequency = 10% Fig. 3C). The most likely candidates for these cells are PVY and PVX. Taken together, these optogenetic data indicate that PVY promotes and sustains backward movement in the context of mating, and that PVX functions as an auxiliary cell that is largely redundant in the circumscribed mating assays used here with Unc hermaphrodites, but which under certain circumstances might enhance PVY-induced motor output.

### PVY+PVX-induced Reversal is Dependent on Activity of AVA

Our cell ablation experiments show that AVA is required for backward movement during the vulva search. If PVY and PVX act through AVA, as the wiring diagram implies [3], then ablation of AVA should block PVY+PVX-induced reversals in our ChR2 assay system. We observed that this was indeed the case. When exposed to a 500 msec flash of blue light, *Pnlp-14::ChR2-YFP* AVA-ablated males were severely impaired in reversal behavior with a significant proportion either pausing, unable to reverse (25%), or reversing a median distance of −1 µm (56%) (Fig. 3B). Thus, PVY+PVX-induced reversal is dependent on AVA function.

AVA interneurons stimulate backward movement by activating the VA and DA backward motor neurons of the ventral nerve cord [15], [20], [21]. Consistent with this, we observed that males defective in VA neuron connectivity (*unc-4* mutants; [34]) were defective in PVY+PVX-induced reversal response and, like AVA-ablated animals, a significant proportion of *unc-4* males either paused (41%) or backed less than a micrometer (27%) (-VA treatment, Fig. 3B). Thus, PVY+PVX-induced backward movement is ultimately dependent on sex-shared backward motor neurons of the ventral cord. Taken together, these experiments delineate a simple neural pathway that consists of the sensory ray neurons, PVY and PVX, AVA and the backward motor neurons.

### PVY+PVX-induced Reversal Depends on Cholinergic Neurotransmission

PVY and PVX express cholinergic markers (Fig. S1A), suggesting that these male-specific interneurons stimulate the command cells using cholinergic transmission. Consistent with this possibility, the command interneurons express several genes encoding cationic *ac*etyl*c*holine *r*eceptor (*acr*) subunits. *acr-15* and *acr-16* encode alpha nicotinic acetylcholine receptor subunits (nAChRs). *acr-15* is expressed in both AVA and AVB, while *acr-16* is expressed only in AVA [35], [36]. We observed that *acr-18,* which encodes a DEG-3 type acetylcholine receptor, is also expressed in AVA, as well as *unc (unc*oordinated*)-29*, which encodes a non-alpha receptor subunit (Fig. S1B, S1C). To test whether any of the cholinergic receptors encoded by these genes are required for PVY+PVX-induced reversal behavior, we artificially activated PVY+PVX in single mutants and in various double and triple mutant combinations of these receptor genes. Mutations in three of these genes (*acr-18*, *unc-29* and *acr-16)* disrupted reversal behavior and are discussed below (for other mutant combinations tested see Fig. S2).

**Figure 4:**
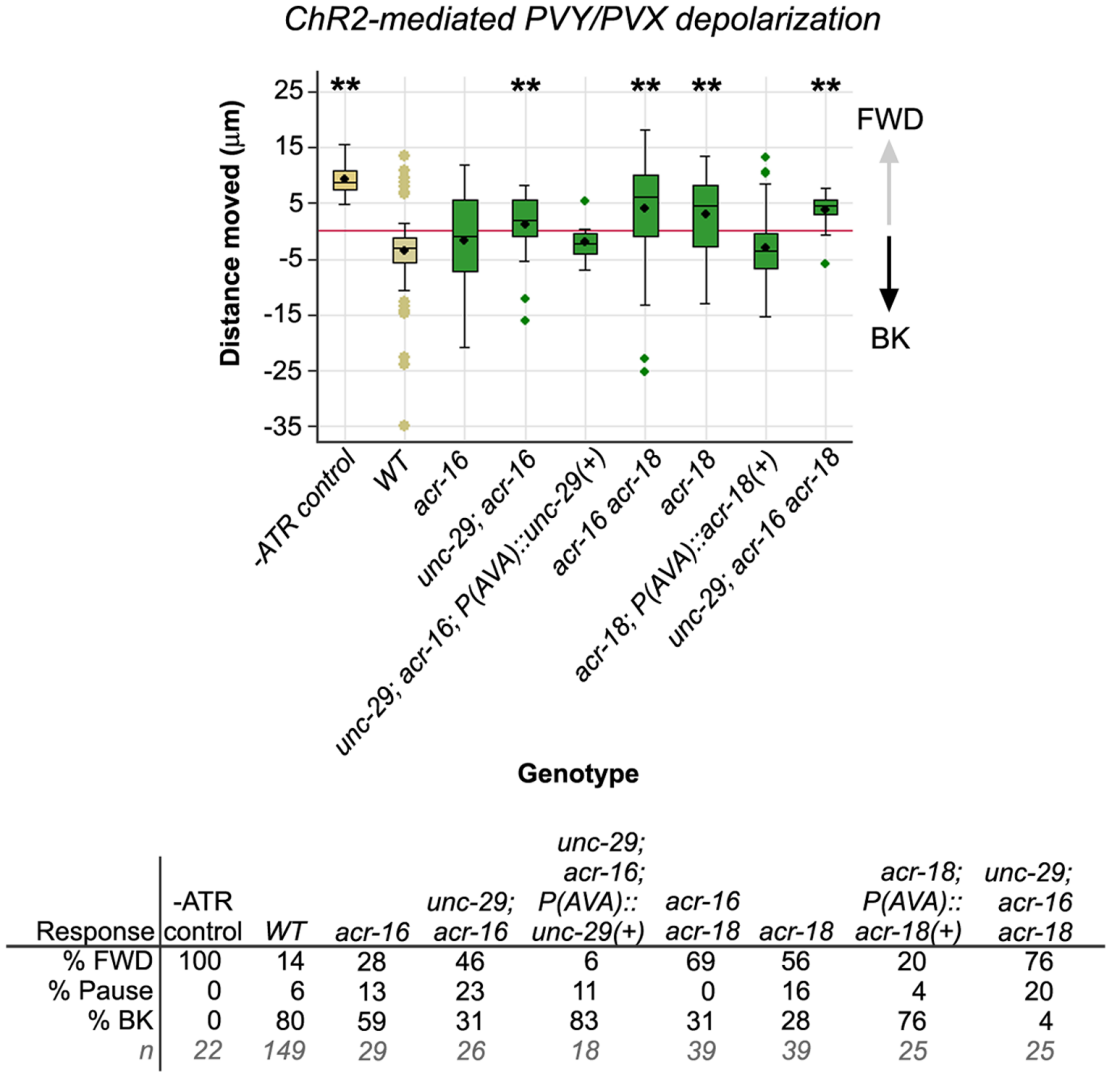
Reversal induced by artificial PVY+PVX activation depends on cholinergic transmission. Males of the genotype indicated on the X-axis and carrying the *Pnlp-14::ChR2-YFP* transgene were subjected to artificial activation assays. See legend for Fig. 3 for details of graph and table format and statistical analyses. Except for the “-ATR control”, all males were cultured and assayed in the presence of ATR. All strains carrying *unc-29* mutations also have the *rgIs1* array which rescues *unc-29* function in body wall muscles. *WT* (wild type) are *him-5* males that are otherwise genetically wild type for the loci tested. Treatments that were statistically different from *WT* are indicated. Significance, *p<0.05; **p<0.005.

In *acr-18* null mutants, only 28% of males reverse in response to PVY+PVX activation (Fig. 4). The remainder either continued to move forward (56%) or paused (16%). *acr-18* reporter expression is not limited to AVA. In both sexes, *acr-18* expression is apparent in a number of sex-shared head and ventral nerve cord neurons and, in the male, in a subset of ray and pre-anal ganglion neurons (Fig. S1B) [37]. To determine whether the absence of *acr-18* function specifically in the backward command cells was responsible for the mutant reversal defect, we targeted expression of a wild type *acr-18* transgene to AVA in *acr-18* mutant males and asked whether this was sufficient to rescue the reversal defect. To this end, we placed a promoterless fragment of the wild type *acr-18* gene (a cDNA::genomic hybrid gene and its 3′ UTR) under the control of the *nmr-1* gene promoter, which is expressed in backward command cells AVA, AVD, AVE and in AVG, RIM and PVC ([38]; Materials and Methods). The *Pnmr-1(AVA)::acr-18(+)* transgene was co-injected with a *Pflp-18::mCherry* construct. *flp-18* is expressed in AVA and we used the presence of mCHERRY in AVA as a proxy for the presence of the *Pnmr-1(AVA)::acr-18(+)* transgene because in *C. elegans,* transgenic arrays typically contain copies of all injected constructs. We observed that the presence of the *acr-18(+)* transgene in the *nmr-1*-expressing cells was sufficient to rescue the *acr-18* mutant defect: 76% of *acr-18* males carrying the rescuing transgene in AVA reversed in response to PVY+PVX activation, whereas only 28% of *acr-18* control males, which lacked this transgene, reversed (Fig. 4). These data suggest that PVY+PVX-induced reversal depends on an ACR-18-containing cholinergic receptor that functions in *nmr-1-*expressing cells, most likely the backward command cells as these are direct targets of cholinergic PVY and PVX.

While the absence of *acr-18* function in AVA significantly impairs reversal behavior in our ChR2 assays, *acr-18* male populations can still reverse at an appreciable frequency (28%). This suggests that other receptors must be involved in mediating transmission. Loss of *acr-16* function alone, or in combination with *acr-18* mutations, caused a modest, but not significant, decrease in reversal frequency, suggesting that *acr-16* contributes to reversal response but is not pivotal (Fig. 4). *acr-16* has been shown to function redundantly with channels containing the non-alpha subunit encoded by *unc-29* in two other tissues in the worm: in the body wall muscles, required for locomotion, and in male-specific muscles that facilitate spicule insertion during copulation [37], [39]. During our analyses of *unc-29; acr-16* spicule insertion behavior, we noticed that double mutant males exhibit reduced scanning speed during the vulva search, a phenotype also exhibited by PVY-ablated males (Fig. 2C) [37], [39]. This prompted us to test whether *unc-29* and *acr-16* might also have partially redundant roles in mediating PVY+PVX-induced reversal behavior. The absence of both *unc-29* and *acr-16* function in body wall muscle renders double mutants severely paralyzed [39]–[41], a defect that precludes mating behavior analyses. However, this locomotion defect can be rescued by expressing an *unc-29* wild type cDNA *(unc-29(+))* specifically in muscles, using the *acr-8* promoter, which is additionally expressed in neurons of the ventral nerve cord [37], [41]. We therefore performed all male behavioral assays involving *unc-29* mutant alleles with strains carrying an integrated version of the muscle-function rescuing transgene, *rgIs1[Pacr-8::unc-29cDNA::SL2::GFP]* [37], [41]. In this transgenic array, *unc-29(+)* is expressed from a polycistronic message that additionally encodes GFP. This provides a convenient means of visualizing *unc-29(+)*-expressing cells as tissues that express GFP should also express *unc-29(+)*. In the PVY+PVX artificial activation assays, the muscle-rescued *unc-29; acr-16* males showed impaired reversal response with only 31% of males reversing (Fig. 4). Thus, *unc-29* loss of function significantly enhances the severity of reversal defects caused by loss of *acr-16* function. As mentioned above, an *unc-29* full-length translational reporter is co-expressed with *acr-16* in AVA and in RIB (Fig. S1C). To test whether the absence of *unc-29* function from AVA might be responsible for the reversal defect of *unc-29; acr-16* males, we performed tissue-specific rescue experiments, targeting expression of *unc-29(+)* to AVA. This was achieved using an *unc-29(+)::SL2::GFP* transgene placed under the control of the *Pnmr-1* promoter. In ChR2-mediated PVY+PVX activation assays, *unc-29; acr-16* males expressing *unc-29(+)* in AVA (indicated by GFP expression in AVA) were rescued, reversing at frequencies comparable to *acr-16* single mutants and wild type males (83%) (Fig. 4). These results suggest that the site of action for *unc-29* in mediating this behavior may be the backward command cells. Given that *unc-29* is partially redundant with *acr-16* in cells were the two genes are co-expressed [37], [39], AVA may also be the site of action *acr-16* in this behavior. As the magnitude of reversals in *unc-29(+)*-rescued double mutants was consistently less than that of *acr-16* single mutant males, *unc-29(+)* may be additionally required in cells downstream of the command cells to enhance the magnitude of backward movement once initiated.

The data above suggest that *unc-29, acr-16* and *acr-18* act in AVA to mediate PVY/PVX-to-AVA cholinergic transmission but are partially redundant. If so, then animals carrying mutations in all three receptor genes should be severely defective in reversal when PVY+PVX are artificially activated. We observed that this was in fact the case: only 4% of *unc-29; acr-16 acr-18* triple mutant males reversed in response to PVY+PVX activation with ChR2 (Fig. 4, Video S7). This mutant phenotype, together with the tissue-specific rescue data, support a model in which PVY/PVX-to-AVA transmission is mediated by three partially redundant AChR subunits.

### Cholinergic Receptor Mutants Exhibit Defects in Locomotion during the Vulva Search

**Figure 5:**
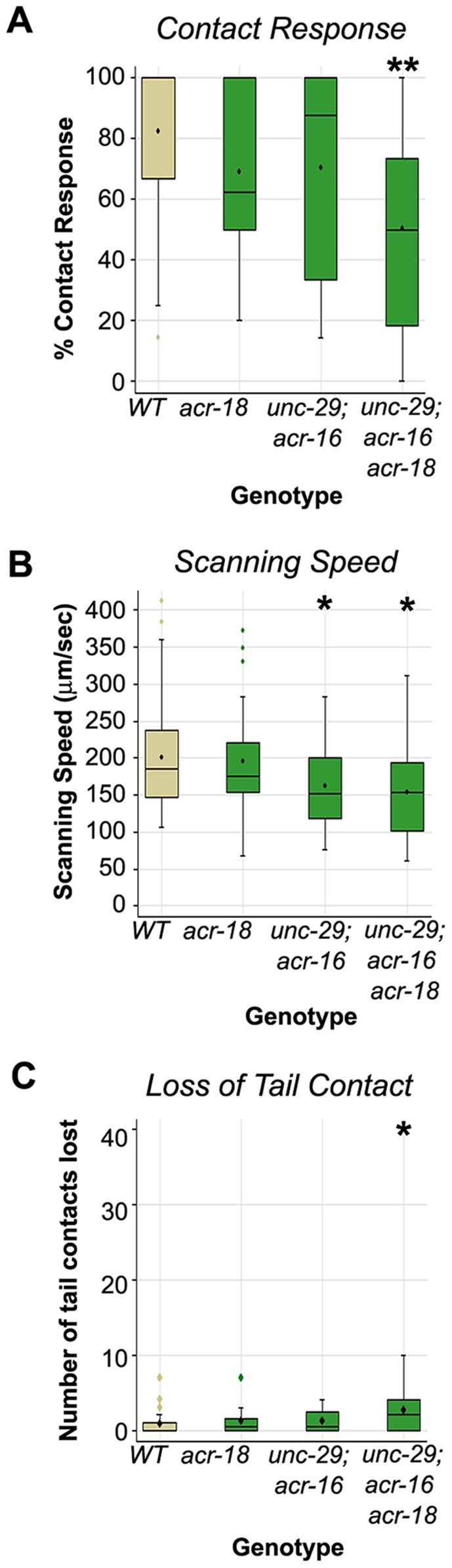
Male movement during mating depends on functionally redundant cholinergic receptors. ***A–C.*** The impact of cholinergic receptor mutations on three aspects of vulva search locomotion. Also see legend for Fig. 2. The X-axis indicates the genetic background examined. All strains carrying *unc-29* mutations also have the *rgIs1* array which rescues *unc-29* function in body wall muscles. *WT* (wild type) are *him-5* males. n: *WT* = 48, *acr-18* = 12, *unc-29; acr-16* = 14, *unc-29; acr-16 acr-18* = 12. Significance, *p<0.05; **p<0.005.

**Figure 6:**
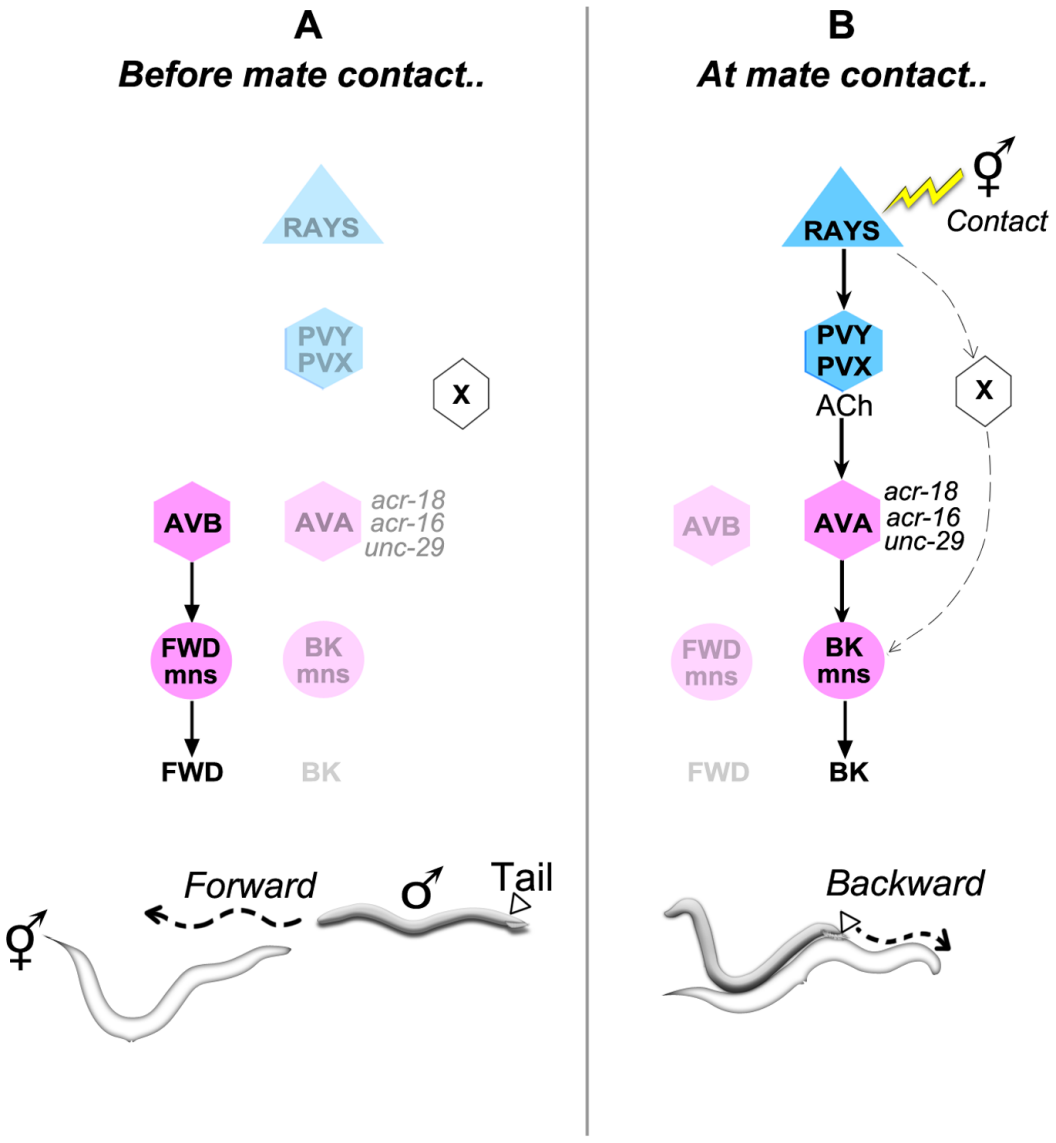
A circuit model for mate contact-induced backward locomotion in the male. A model for how mate contact alters the activity of the sex-shared locomotory system to induce directional change. Shown are the activity states of circuit components in the absence *(A)* or the presence of mate contact *(B),* with the corresponding male behavior depicted below. Cell type and sex-specificity is indicated by the symbol and color, respectively: sex-shared cells (pink), male-specific cells (blue), sensory neurons (triangles), interneurons (hexagons), motor neurons (circles). Color intensity indicates a cell activity state (intense color = high; weak color = low). The arrows indicate the positive action of an activated cell on its postsynaptic target. ***A.*** When not engaged in mating, the male moves with a forward directional bias due to high levels of activity in the forward pathway of the sex-shared locomotory system: forward command interneurons AVB and their motor neuron targets (FWD mns). This activity bias is conferred by the default state of this system [21], in the absence of specific cues, or by external or internal sensory cues that drive the male to explore his environment. ***B.*** Ray neuron stimulation by mate contact activates PVY and PVX, which release acetylcholine (ACh), preferentially stimulating the backward (AVA), and not forward, command interneurons. This is due to a bias in synaptic inputs and in cholinergic receptor expression (ACR-18, ACR-16 and UNC-29) in favor of the backward command cells. AVA activation in turn stimulates the sex-shared backward motor neurons (BK mns). The rays may also promote backward movement through an as yet uncharacterized PVY/PVX-independent pathway (represented as interneuron “X”).

We next asked whether disruption of PVY/PVX circuit function in AChR mutants affected search behavior in the context of mating. Surprisingly, we found that although more than 50% of *acr-18* single mutants are defective in reversal behavior in the ChR2 assays, *acr-18* males were superficially wild type for backward locomotion in the context of mating (Fig. 5). *unc-29; acr-16* double mutants also performed this behavior reasonably well although showed reduced scanning speed as previously reported [37] (median scanning speed = 150 µm/sec *cf.* wild type control male median = 180 µm/sec, Fig. 5). The fact that *unc-29; acr-16* mutants are affected in vulva search locomotion speed, and *acr-18* mutants are not, suggests that *unc-29* and *acr-16* encoded channels may be more critical in determining locomotory speed than the *acr-18* encoded channel. The fact that neither *acr-18* single- nor *unc-29; acr-16* double mutants resembled PVY-ablated males (Fig. 2) suggests that the male nervous system can compensate for the partial disruption of PVY-dependent transmission, possibly through upregulation of the remaining functional receptors in these respective mutant backgrounds. Consistent with this possibility, males mutant for all three receptor genes (*unc-29; acr-16 acr-18* triple mutants) were significantly affected in all aspects of vulva search locomotion that we measured (Fig. 5; Video S8). The triple mutant defects were, in fact, similar to those displayed by males lacking PVY and PVX (Fig. 2). *unc-29; acr-16 acr-18* mutants had a median contact response efficiency of 50% (*cf.* 50% for the -PVY-PVX treatment, Fig. 2), a median scanning speed of 140 µm/sec (*cf.* 110 µm/sec for the -PVY-PVX treatment) and a median loss of contact frequency of 2 (*cf.* 8 for the -PVY-PVX treatment) (Fig. 2, Fig. 5). The similarity of the triple mutant to cell-ablated males is consistent with the ChR2 assay data, which argues that eliminating all three receptors essentially blocks PVY+PVX-induced backward movement. Like -PVY-PVX males, the triple mutant can still reverse in the context of mating, albeit inefficiently, suggesting that other ray-targeted pathways can partially compensate for the absence of PVY/PVX pathway activity and promote backward movement (see model in Fig. 6).

## Discussion

How animals integrate and prioritize external cues and select the most adaptive behavioral response is a broadly relevant question. Sensory control of directional movement in *C. elegans* provides a tractable system for exploring this issue and has been the subject of intense investigation in the hermaphrodite. Here we examine this issue in the context of a sex-specific behavior and ask: how do mate contact cues override the forward locomotion bias of exploratory behavior and induce the male to pursue sex? Mate contact, sensed by the rays, causes the male to abruptly switch from forward to backward movement in order to conduct a systematic, contact-based search for the vulva. Our data suggest a simple circuit model to explain how ray stimulation by mate contact overrides a bias for forward movement, set by intrinsic activity of the sex-shared locomotory system or induced by external cues that promote exploration. In this model, ray neuron stimulation preferentially upregulates the activity of the backward command cells and consequently resets the activity patterns of locomotory motor neurons in favor of backward movement (Fig. 6). Ray control of the sex-shared command interneurons is primarily mediated via the male-specific PVY interneuron. Both the hard wiring of this ray-to-command cell pathway and the distribution of key molecular components within it would be predicted to induce a high probability of reversal in response to mate contact. First, the wiring diagram argues that the rays, PVY/PVX and the command cells form a feedforward pathway, as there are virtually no reciprocal connections between the rays and PVY/PVX or between PVY/PVX and AVA. Second, although PVY and PVX have inputs into both forward and backward command cells, this connectivity is biased in favor of AVA. Third, the key receptors that mediate cholinergic neurotransmission in this pathway (encoded by *acr-18, unc-29* and *acr-16)* appear to be preferentially expressed on the backward command cells.

Interestingly, we find that removal of PVY, PVX and even AVA does not completely abolish reversal in the context of mating, indicating that although these cells are important for this behavior, other ray neuron targets can partially compensate for their absence. The interneuron “X” in Fig. 6 denotes this hypothetical, alternative pathway. The ray neurons make connections with several sex-shared interneurons that have inputs onto the command cells and could potentially fulfill this role (PVN, AVH, AVJ and AVF) [3]–[5]. It is also possible that this putative pathway corresponds to the disinhibitory pathway, a second directional control system that operates in parallel with the command cells in certain behavioral contexts [25]. The use of parallel pathways would be predicted to confer behavioral robustness and, if these pathways have subtly different roles, behavioral acuity. In the mating assays performed here we used Unc mating partners, which are relatively docile. However, wild type hermaphrodites are less compliant and dart or continue moving during the search [27], making it necessary for the male to adjust his speed and direction in order to maintain tail contact. In this context, robust and accurate control of movement is essential for mating success.

By several criteria, PVY corresponds to a decision-making neuron of the male nervous system. Its artificial activation in the absence of upstream ray stimulation is sufficient to induce backward locomotion in solitary males. Conversely, its hyperpolarization or elimination significantly impairs movement on the hermaphrodite in the context of mating. Moreover, PVY and PVX receive inputs from several tail sensilla [3], both male-specific (the rays, the hook and the p.c.s.) and sex-shared (the phasmids), consistent with the possibility that PVY (and PVX) represent a site of sensory stimulus integration and that the outcome of this integration determines whether contact-induced reversal is initiated, maintained or terminated. Recent studies show that phasmid and other sex-shared sensory neurons contribute to mate-searching behavior, which is conducted with a forward directional bias [42]. Potentially, the substantial inputs from these sensory cells onto PVX may serve to suppress PVY+PVX-dependent reversal until a mate is located. During mating, detection of the vulva by the male hook and p.c.s. induces the male tail to pause at the vulva and remain there during ensuing copulatory attempts [11]. A simple model would be that the hook neurons induce pausing by inhibiting PVY and PVX activity. This would result in suppression of backward movement without affecting tail apposition. Consistent with this possibility, hook neurons have inputs onto PVY and PVX [3]. However, these inputs are surprisingly sparse compared to those made by hook neurons with their other targets. These latter postsynaptic targets include several neurons with inputs onto the command interneurons or ventral nerve cord motor neurons (*e.g.*, sex-shared AVG and the male-specific EF and PVZ interneurons). Whether the hook induces pausing by inhibiting PVY/PVX directly or indirectly is therefore an open question. Future studies, examining the dynamics of PVY, PVX and command cell activity in these various behavioral contexts should provide insight into how converging sensory pathways alter directional movement through their action on PVY and PVX.

PVY+PVX-induced reversal depends on cholinergic transmission in AVA that is mediated by partially redundant AChRs that contain *acr-18-, unc-29-* and *acr-16-*encoded subunits. The existence of AChRs in the command cells was initially revealed in studies examining the impact of exogenous nicotine on worm behavior [35]. However, to our knowledge, our study is the first to demonstrate a role for command cell cholinergic transmission in a natural *C. elegans* behavior. While the command cells have a few inputs from sex-shared cholinergic cells (*e.g.*, the PVC forward command interneurons, the SDQ interneurons of the oxygen sensing pathway and tail interneuron DVC [43], [44]), many inputs are from glutamatergic neurons and these regulate the command cells through cationic glutamatergic receptors [25], [45]–[47]. The use of cholinergic transmission in the male may therefore contribute to the specificity of command system control by the male nervous system. It could also serve to coordinate reversal with other motor outputs associated with mating that depend on cholinergic signaling, such as tail apposition and spicule prodding behavior [14], [48]. The rays induce tail apposition in part through cholinergic stimulation of tail muscles [48], [49] and, during vulva penetration attempts, cholinergic neurons of the p.c.s. may further potentiate ray action to cup the tail over the vulva [37]. Tail apposition posture depends on sex-shared muscles that are normally engaged in wave propagation. Therefore, the absence of wave propagation in the tail during mating may be simply due to the fact these muscles are otherwise engaged in tail apposition, as a consequence of ray cholinergic transmission. The predominance of cholinergic signaling in mating behavior circuits may also relate to the rapid speed with which the male must respond to hermaphrodite surface cues, as cholinergic transmission also features in rapidly executed behaviors in other invertebrate systems, such as escape response in *Drosophila*, crickets and snails [50]–[52]. In these latter systems, the sensory neurons are cholinergic and act on giant fibers. Giant fibers, in turn, are electrically coupled to their motor neuron targets, a design feature that likely contributes to rapid response. The *C. elegans* command interneurons could be considered analogous to giant fibers. Like giant fibers, their processes extend long distances and regulate an extensive population of motor neurons, notably through gap junction connections [7].

Although behavioral sexual dimorphism is widely observed in the animal kingdom, identifying their neural and molecular bases in many systems is hindered by genomic intractability and nervous system complexity. Functional dissection of sexual behaviors in simple, invertebrate models has provided some insights into where and how pivotal sex differences in nervous system function are encoded and to what extent cells common to both sexes are employed. In *C. elegans*, sex-shared cells contribute to several male-specific behaviors. For example, the sex-shared DVA neuron is the source of oxytocin/vasopressin-like peptides that enhance the robustness and coherence of mate-searching and mating sub-behaviors by priming activity of the underlying circuitry [53]. Sex-shared sensory pathways also provide critical input for mate-searching behavior, mate attraction, male-specific odor preference and aspects of vulva search behavior [42], [54]–[58]. Although sex-shared sensory pathways are required for mate attraction and male odorant preference, sexually dimorphic properties of these neurons confer the male-specificity of the response [54], [57]. In contrast, we find that a sensory pathway composed of strictly male-specific cell types is primarily responsible for inducing male-specific vulva search behavior. The presence of this ray-PVY/PVX pathway in the male renders the sex-shared locomotory system responsive to genital contact with a hermaphrodite. It does so by targeting key control centers in the sex-shared locomotory system, namely AVA. In the *C. elegans* hermaphrodite a similar circuit design underlies the locomotory patterns associated with egg-laying. In this case, the Hermaphrodite-Specific motor Neurons (HSNs) of the egg-laying system cause a burst of forward acceleration to coincide with egg release via direct regulation of sex-shared locomotory system interneurons [59]. Similarly, in *Drosophila* the reason males produce a courtship song using wing vibrations, and females do not, is primarily due to male-specific modulation of sex-shared central pattern generators for wing vibration [60]–[62]. These various examples, along with our own interrogation of the PVY/PVX pathway, provide insight into how new reproductive behaviors might evolve through relatively simple modifications to shared circuitry. Circuit modifications of these types may make prominent contributions to natural variations in behavior that ultimately bring about speciation.

## Materials and Methods

### Strains

*unc-29(e193)I* [63], [64], *unc-64(e246)III* [9], *pha-1(e2123ts)III* [65], *acr-15(ok1214)V*, *acr-16(ok789)V*, *acr-18(ok1285)V* (*C. elegans* Gene Knockout Consortium), *him-5(e1490)V* [66], *nlp-14(tm1880)X* (National BioResource Project), *lite-1(ce314)X* [31], *akIs3[Pnmr-1::GFP+lin-15(+)]V* [38], *rgIs1[Pacr-8(muscle)::unc-29(+)::SL2::GFP], rgEx387[Punc-29::unc-29::YFP+pha-1(+)], rgEx196[Pacr-18:ChR2::YFP+ pha-1(+)]* [37]. All strains used in this study carried the *him-5(e1490)* mutation which generates a *h*igh *i*ncidence of *m*ales [66]. *pha-1(e2123ts)* was grown at 15°C. All other strains were maintained at 20°C and cultured as per Brenner (1974) [9].

### Transgenic Arrays

*fkEx32, fkEx77: Ex[Pnlp-14(PVY+PVX)::ChR2-YFP+Punc-122::GFP].*

*fkEx63: Ex[Pnlp-14(PVY+PVX)::mCherry+Pttx-3::mCherry].*

*fkEx66, fkEx67: Ex[Pnlp-14(PVY+PVX)::NpHR-EYFP+pha-1(+)].*

*fkEx76: Ex[Pnmr-1(AVA)::mCherry+Punc-122::GFP].*

*fkEx71: Ex[Pflp-18(AVA)::mCherry+Pttx-3::GFP].*

*fkEx72: Ex[Pflp-18(AVA)::mCherry+Pnlp-14(PVY-PVX)::ChR2-YFP].*

*fkEx92, fkEx93: Ex[Pnmr-1(AVA)::acr-18(+)+flp-18(AVA)::mCherry].*

*fkEx94: Ex[Pacr-16::mCherry+Pttx3::mCherry].*

*fkEx94, fkEx95: Ex[Pnmr-1(AVA)::unc-29(+)::SL2::GFP+Pttx-3::mCherry].*

Transgenic lines were generated using standard microinjection techniques [67]. Constructs were injected at the following concentrations: *Pnlp-14::ChR2-YFP* (100 ng/µL); *Pflp-18::mCherry* (40 ng/µL), *Pacr-16::mCherry* (100 ng/µL); *Pnmr-1(AVA)::acr-18(+)* (50 ng/µL), *Pnmr-1(AVA)::unc-29(+)::SL2::GFP* (50 ng/µL). Co-transformation markers, pBX-1*(pha-1(+)), Pttx-3(AIY)::mCherry*, *Pttx-3(AIY)::GFP* and *Punc-122(coelomocytes)::GFP* were each injected at 50 ng/µL.

### DNA Constructs

All plasmids used in this study were generated using the Gateway cloning system (Invitrogen). To make the entry vectors, promoter fragments were PCR amplified from genomic or plasmid DNA templates using gene-specific promoter primers containing attB1 and attB2 sequences. The gene-specific sequences for these primers are as follows:

*nlp-14* promoter (based on Nathoo et al. (2001) [30]).

FWD: GTTTACCCAGCTTTTTTCATTGTAGAAAACATCAC.

REV: TGTGCGTGTGTTACCCGGAAAG.

*flp-18* promoter.

FWD: GCAAATCTGTCACATACTGCTCGAATCG.

REV: ACCGTTGCATGTCTAACCCTGAAATTATTA.

*acr-16* promoter (based on Feng et al. (2006) [35]).

FWD: GATCCGAGAACATGACGATGACAATGATG.

REV: TACGGACATGAGAATCAGGGAAAGAAAAGC.

*nmr-1* promoter (based on Brockie et al. (2001) [38]).

FWD: GACACTTTCATCTGTTCAGAATTGAGATGC.

REV: AACTAAAGTTTGTCGTGTTCCAAACAGAAG.

PCR fragments were then cloned into pDONR221 using BP clonase to generate the entry vectors. Entry vectors were then recombined with the appropriate destination vector using LR clonase II. Destination vectors used in this study were *ccdB C.1::ChR2-YFP* (pLR167) [12], *ccdB C.1::NpHR-EYFP* (pZL19) *version 2, ccdB (C.1)::mCherry* (pZL19), *ccdB C.1 unc-29(+)::SL2::GFP*) (pYL16) [37]. To generate *ccdB C.1::NpHR-EYFP,* a fragment containing *eNpHR3.0-EYFP* was generated by PCR using *pLenti-hSyn-eNpHR3.0-EYFP* [32], [33] (http://www.stanford.edu/group/dlab/optogenetics/sequence_info.html) as the template and primers containing *BamH1* and *EcoR1* ends. After restriction digestion, this fragment was exchanged with the *ChR2-YFP BamH1/EcoR1* fragment in *Pmec-4::ChR2-YFP* [29]. Next, the *mec-4* promoter was removed by digesting with *HindIII* and *BamH1*. The vector fragment ends were filled, phosphatase-treated, then ligated with the ccdB C.1 cassette. This insertion created a stop codon (TGA) at the cassette/vector junction, which was changed to a glycine codon (GGA) using site-directed mutagenesis to generate pZL19. In the *acr-18* tissue-specific rescue experiments, a fragment containing the *acr-18* wild type open reading frame and its 3′ UTR (generated by PCR fusion of a partial cDNA sequence and genomic DNA) was fused to an *nmr-1* promoter by PCR based on the method of Hobert (2002) [68]. The *Pnmr-1::unc-29(+)::SL2::GFP* construct was generated by recombining the *Pnmr-1*-containing entry vector with the destination vector pYL16 in a LR clonase reaction.

### Laser-mediated Cell Ablations

Laser-mediated cell ablations (for data presented in Fig. 2 and Fig. 3) were performed on L4 transgenic males using standard procedures [69]. Control (mock-ablated) L4 males were mounted with anesthetic for the same duration but not operated on. Males were allowed to mature overnight into adults, isolated from hermaphrodites. For males requiring ChR2 assays, the OP50 food was supplemented with 50 µM ATR. Males were then subjected to standard mating assays and/or ChR2 assays (described below). To confirm that targeted cells had been killed in operated animals, each male was mounted on a slide after assays and examined at 600× magnification on a Zeiss D1 compound microscope equipped with epi-fluorescence. AVA ablations were performed on males carrying the transgenic arrays *fkEx71* or *fkEx72*; PVY and/or PVX ablations were performed on strains carrying *fkEx32* or *fkEx77*.

### Mating Behavior Assays

Mating assays (for Fig. 2, Fig. 3C and Fig. 5) were carried out based on procedures described in Liu et al. (2007) [55]. Twenty-four hours before assays, L4 males (maximum of 10) were placed together on a culture plate and allowed to mature overnight in the absence of hermaphrodites. The next day, individual males were placed on a mating lawn (a 5 mm diameter OP50 lawn, freshly prepared) containing 5 *unc-64; lite-1* virgin 1-day old adult hermaphrodites. Their mating behavior was observed for 15 mins or until the male ejaculated, whichever occurred first. Each assay was digitally recorded using a Zeiss AxioCam HS digital camera and AxioVision software (release 4.7). These videos were analyzed for execution of the three motor behaviors presented in Fig. 2 and Fig. 5: % of successful contact responses, scanning speed on the non-vulva side and the frequency of lost tail contact during scanning. % Contact Response = 100 x [the number of times the male exhibits contact response/the number of times the male makes contact with a hermaphrodite via the rays]. A successful (complete) contact response requires both tail apposition and initiation of backward locomotion [12]. Scanning speed on the non-vulva side (µm/sec) = mean length of 1-day old adult *unc-64; lite-1* hermaphrodites (*i.e.,* 1044 µm)/the time required for a male to travel the length (sec). The average speed for a male was calculated from a random selection of 5 non-vulva sides scanned, or all non-vulva sides scanned if the number of sides completed was less than 5. Number of tail contacts lost = the number of times that a male lost tail contact with the hermaphrodite during the mating trial.

### ChR2 and NpHR Assays

Relates to Fig. 3, Fig. 4 and Fig. S2. Except for –ATR controls, all strains were maintained on plates spread with OP50 *E. coli* supplemented with 50 µM ATR. Except during animal transfer or assays, plates were kept wrapped in foil. Twenty-four hours before assaying, five L4 males were placed on a plate freshly spread with 50 µM ATR+OP50 and allowed to mature overnight. Assays were performed on a Zeiss M2 Imager stereomicroscope equipped with epi-fluorescence. For the ChR2 assays, individual animals were exposed to three, evenly spaced, 500 msec flashes of blue light (470/40 nm wavelength), while moving forward. Assays were recorded using a Zeiss AxioCam HS camera and AxioVision software [12]. For PVY/PVX activation, strains carrying either array *fkEx32*, *fkEx72 or fkEx77* were used. The presence of ChR2-YFP in both PVY and PVX was verified post-assay at 600× magnification on a Zeiss D1 compound microscope equipped with epi-fluorescence.

To inhibit PVY and PVX during mating, strains of the following genotype were used*: fkEx66 or fkEx67 [Pnlp-14(PVY+PVX)::NpHR-EYFP+pha-1(+)]; pha-1(e2123ts); him-5(e1490)V; lite-1(ce314)X.* Strains were cultured with or without ATR as per ChR2 transgenic strains (+ATR and –ATR treatments, respectively in Fig. 3C). Twenty-four hours before assaying, five L4 transgenic males were placed on a plate freshly spread either with or without ATR. The next day, individual males were placed on a mating lawn with 5 *unc-64; lite-1* 1-day old virgin hermaphrodites. Males were allowed to initiate the vulva search with contact response and commence scanning. Males were then exposed to a 500 msec pulse of yellow light (540/25 nm) at 5 evenly spaced time intervals during scanning. The percentage of times a male paused when pulsed with light was scored. To test the effect of NpHR activation in transgenic males not engaged in mating, 1-day old, solitary virgin transgenic males (grown in the presence or absence of ATR) were exposed to three, evenly spaced 500 msec yellow light flash, while they were moving forward. The distance and direction travelled after the flash was measured as described below.

### acr-18 and unc-29 Tissue-specific Rescue Experiments

*acr-18* rescue: *acr-18; fkEx92 or fkEx93 [Pnmr-1(AVA)::acr-18(+)+Pflp-18(AVA)::mCherry]* hermaphrodites were mated with *acr-18; fkEx32 or fkEx77[nlp-14(PVY+PVX)::ChR2-YFP+Punc122::GFP]* males. F1 L4 male progeny that were both UNC-122::GFP- and mCHERRY-positive, were transferred to ATR-containing plates (5 males per plate) then allowed to mature to adulthood overnight. Individual males were then subjected to ChR2 assays. After each male was assayed, the animal was mounted on a slide and examined for CHR2-YFP expression in both PVY and PVX and for the presence of mCHERRY in AVA at 600× or 400× magnification. Males that were mChERRY-positive in AVA and ChR2-YFP-postive in both PVY and PVX correspond to the “*acr-18; P(AVA)::acr-18(+)*” treatment, Fig. 4.

*unc-29 rescue: unc-29; acr-16; rgIs1[Pacr-8(muscle)::unc-29(+)::SL2::GFP]*; *fkEx95 or 96[Pnmr-1(AVA)::unc-29(+)::SL2::GFP+Pttx-3::mCherry]* hermaphrodites were mated with *unc-29; acr-16; rgIs1[Pacr-8(muscle)::unc-29(+)::SL2::GFP]; fkEx32* males. F1 progeny positive for both *fkEx32* and *fkEx95/96* arrays were subjected to ChR2 assays and handled as per males in the *acr-18* rescue experiments. Males that expressed ChR2-YFP in both PVY and PVX correspond to the “*unc-29; acr-16; P(AVA)::unc-29(+)*” treatment, Fig. 4.

### Measurement of the Distance Moved in Response to ChR2-mediated PVY+PVX Activation

Animal response after ChR2 activation was captured digitally as described above. The frame after the light flash in which response is first apparent (“start of response” frame) was identified as well as the frame in which response ended (“end of response” frame). In each frame, the animal’s position in the frame was determined by measuring the distance from the animal’s head (or tail) to the closest frame edge. The “distance moved” (in µm) = the position in the “start of response” frame – the position in the “end of response” frame, where a negative value corresponds to backward movement and a positive value corresponds to forward movement. Although each animal was exposed to three flashes, the first flash usually gave the most unambiguous response and was typically used for analysis. For animals that did not respond to the light flashes, the distance moved was determined using the time frames that corresponded to the average “start of response” and “end of response” times for the positive controls (in Fig. 3A this was M; in Fig. 3B, Control; in Fig. 4, wild type).

To calculate the % of males that backed, paused or moved forward for each population assayed in Fig. 3, Fig. 4 and Fig. S2, backing, pausing or forward movement were classified by the following criteria: backing = distance travelled is <0 µm; pausing = distance is 0 to +2.5 µm; forward = distance>+2.5 µm.

### Statistical Analysis

The following variables were measured:

1. Mating Behavior Assays:
  - % Contact response (Fig. 2B, Fig. 5A)
  - Scanning speed (µm/sec) (Fig. 2C, Fig. 5B)
  - Number of tail contacts lost (Fig. 2D, Fig. 5C)
2. Directional movement in ChR2 and NpHR assays:
  - Locomotion Assays - Distance moved (µm):
    - Ablated animals and mock controls (Fig. 3A, 3B)
    - Mutants (Fig. 4, Fig. S2)
  - Mating assays - % of times each male paused in response to a light flash (Fig. 3C).

All groups of observations for each measurement were tested for normality using the Shapiro-Wilks test. Most were found to be not normal, thus the Wilcoxon-Mann-Whitney.

ranksum test was used to compare two populations. Since up to 5 speeds per male were used in the scanning speed measurements, significance was determined by clustered bootstrapping on the difference between the observed and expected ranks.

## Supporting Information

Figure S1 **PVY and PVX are cholinergic and their command interneuron targets express cholinergic receptor genes.** ***A.*** Fluorescent micrograph of an adult male tail showing co-expression of a *Pnlp-14::*mCHERRY reporter and a cholinergic marker (UNC-17::GFP) [14], [44] in PVY and PVX. The dotted line indicates the approximate boundary of the pre-anal ganglion (PAG) in which PVY and PVX reside. The spicules (SP) are visible due to their auto-fluorescent properties. ***B.*** Fluorescent micrograph of an L3 male showing expression of a *Pacr-18::*ChR2-YFP transgene in head neurons, including AVA. This reporter is also expressed in neurons of the ventral nerve cord (VNC) in both sexes and in a subset of ray and PAG neurons in the male (data not shown). ***C.*** Fluorescent micrograph of an L3 male showing expression of a full-length *unc-29* translational reporter in neurons of the head. *, cells in which *unc-29* and *acr-16* reporters are co-expressed. The expression pattern of the *unc-29* reporter in the hermaphrodite is superficially similar to that of the male. SLs in (*B)* and (*C)* correspond to sub-lateral neurons (possibly SMB/Ds, SIBs or SIAs). For all images the scale bar indicates 10 µm.
(TIF)

Figure S2 **The impact of** ***acr-15*** **and** ***nlp-14*** **mutations on PVY+PVX-induced reversal behavior.** Males of the genotype indicated on the X-axis and carrying the *Pnlp-14::ChR2-YFP* transgene were subjected to artificial activation assays. See legend of Fig. 3 for details of graph and table format and statistical analyses. Except for the “-ATR control”, all males were cultured and assayed in the presence of ATR. *acr-15* has no significant impact on reversal behavior, either as a single mutation or in combination with *acr-18* mutations, arguing that the absence of phenotype in *acr-15* single mutants is not a consequence of functional redundancy with *acr-18*. In addition to cholinergic markers, PVY and PVX express the *nlp(n*europeptide*-l*ike *p*rotein*)-14* gene, which is predicted to encode neuropeptides with sequence similarity to orcokinin from *Orconectes limosus* (crayfish) [30]. *nlp-14* mutants show impaired reversal response. *acr-18; nlp-14* double mutants are phenotypically similar to *acr-18* single mutants. This suggests that NLP-14 has a neuromodulatory role in PVY/PVX transmission and that cholinergic signaling (mediated by ACR-18- ACR-16- and UNC-29-containing receptors) is the rate-limiting factor. Treatments that were statistically different from wild type (*WT*) are indicated. Significance, *p<0.05; **p<0.005.
(TIF)

Video S1 ***C. elegans*** **male locomotion during the vulva search of mating has a backward directional bias.** Shown is a control (wild type) male executing all of the motor behaviors that make up the vulva search: contact response, scanning and turning. Related to Fig. 2.
(MOV)

Video S2 **Males lacking the male-specific interneuron PVY are defective in vulva search locomotion.** This PVY-ablated male fails to respond to contact, is unable to maintain backward movement and tail contact and exhibits slow backward locomotion. Related to Fig. 2.
(MOV)

Video S3 **ChR2-mediated depolarization of PVY+PVX induces reversal in solitary males.** Shown is the reversal response of a transgenic male (grown in the presence of ATR) expressing ChR2 in the PVY+PVX neurons (genotype: *fkEx32[Pnlp-14(PVY+PVX)::ChR2-YFP+Punc-122::GFP]; him-5(e1490); lite-1(ce314)*). At the movie start, the tail is at the bottom of the frame and the head at the top. The fluorescent spots on the body that appear during the light flash correspond to GFP expression in the coleomocytes (produced by the *Punc-122::GFP* co-transformation marker). The movie is played at reduced speed for clarity. Related to Fig. 3.
(MOV)

Video S4 **A control male, expressing non-functional ChR2 in PVY+PVX, does not alter his locomotion in response to blue light.** Shown is a transgenic control male of the same genotype as the male in Video S3 (genotype: *fkEx32[Pnlp-14(PVY+PVX)::ChR2-YFP+Punc-122::GFP]; him-5(e1490); lite-1(ce314)),* but grown in the absence of ATR. The tail is to the right; head to the left. The fluorescent spots on the body that appear during the light flash correspond to GFP expression in the coleomocytes (produced by the *Punc-122::GFP* co-transformation marker). The movie is played at reduced speed for clarity. Related to Fig. 3.
(MOV)

Video S5 **NpHR-mediated hyperpolarization of PVY+PVX induces pausing during the vulva search.** Shown is a transgenic male, grown in the presence of ATR, expressing functional NpHR in PVY+PVX neurons (genotype: *fkEx66[Pnlp-14(PVY+PVX)::NpHR-EYFP+pha-1(+)]; pha-1(e2123ts); him-5(e1490); lite-1(ce314)*). The male pauses in response to a pulse of yellow light (which induces NpHR-mediated hyperpolarization of PVY and PVX). Related to Fig. 3.
(MOV)

Video S6 **A control male, expressing non-functional NpHR in PVY and PVX does not alter his locomotion during mating in response to yellow light.** Shown is the behavior of a transgenic male, grown in the absence of ATR, expressing non-functional NpHR in PVY and PVX neurons (genotype: *fkEx66[Pnlp-14(PVY+PVX)::NpHR-EYFP+pha-1(+)]; pha-1(e2123ts); him-5(e1490); lite-1(ce314)*). Related to Fig. 3.
(MOV)

Video S7 ***unc-29; acr-16 acr-18*** **males fail to reverse in response to ChR2-mediated PVY+PVX activation.** Shown is a triple mutant male expressing functional ChR2 in PVY+PVX (array *fkEx32*). In contrast to wild type (WT) control male behavior (Video S3), the triple mutant fails to reverse in response to the light flash and continues forward locomotion. The tail is to the right; head to the left. The fluorescent spots on the body that appear during the light flash correspond to GFP expression in the coleomocytes (produced by the *Punc-122::GFP* co-transformation marker). The movie is played at reduced speed for clarity. Related to Fig. 4.
(MOV)

Video S8 ***unc-29; acr-16 acr-18*** **males exhibit directional movement defects in vulva search behavior.** The triple mutant males have similar locomotion defects to PVY-ablated males (Video S2). Related to Fig. 5.
(MOV)

## References

[1] Williams TM, Carroll SB (2009) Genetic and molecular insights into the development and evolution of sexual dimorphism. Nat Rev Genet 10: 797–804. 19834484 10.1038/nrg2687

[2] Dickson BJ (2008) Wired for sex: the neurobiology of *Drosophila* mating decisions. Science 322: 904–909. 18988843 10.1126/science.1159276

[3] Jarrell TA, Wang Y, Bloniarz AE, Brittin CA, Xu M, et al (2012) The connectome of a decision-making neural network. Science 337: 437–444. 22837521 10.1126/science.1221762

[4] Sulston J, Albertson D, Thomson J (1980) The *Caenorhabditis elegans* male: Postembryonic development of nongonadal structures. Dev Biol 78: 542–576. 7409314 10.1016/0012-1606(80)90352-8

[5] Sulston J, Horvitz H (1977) Post-embryonic cell lineages of the nematode, *Caenorhabditis elegans* . Dev Biol 56: 110–156. 838129 10.1016/0012-1606(77)90158-0

[6] Hall D, Russell R (1991) The posterior nervous system of the nematode *Caenorhabditis elegans*: Serial reconstruction of identified neurons and complete pattern of synaptic interactions. J Neurosci 11: 1–22. 1986064 10.1523/JNEUROSCI.11-01-00001.1991 PMC6575198

[7] White J, Southgate E, Thomson J, Brenner S (1986) The structure of the nervous system of the nematode *Caenorhabditis elegans* . Phil Trans Royal Soc London Series B, Biol Scien 314: 1–340. 10.1098/rstb.1986.0056 22462104

[8] Xu X, Kim SK (2011) The early bird catches the worm: new technologies for the *Caenorhabditis elegans* toolkit. Nat Rev Genet 12: 793–801. 21969037 10.1038/nrg3050 PMC4719766

[9] Brenner S (1974) The genetics of *Caenorhabditis elegans* . Genetics 77: 71–94. 4366476 10.1093/genetics/77.1.71 PMC1213120

[10] Barker DM (1994) Copulatory plugs and paternity assurance in the nematode *Caenorhabditis elegans* . Anim Behav 48: 147–156.

[11] Liu KS, Sternberg PW (1995) Sensory regulation of male mating behavior in *Caenorhabditis elegans* . Neuron 14: 79–89. 7826644 10.1016/0896-6273(95)90242-2

[12] Koo PK, Bian X, Sherlekar AL, Bunkers MR, Lints R (2011) The robustness of *Caenorhabditis elegans* male mating behavior depends on the distributed properties of ray sensory neurons and their output through core and male-specific targets. J Neurosci 31: 7497–7510. 21593334 10.1523/JNEUROSCI.6153-10.2011 PMC6622613

[13] Barr MM, Sternberg PW (1999) A polycystic kidney-disease gene homologue required for male mating behaviour in *C. elegans* . Nature 401: 386–389. 10517638 10.1038/43913

[14] Garcia LR, Mehta P, Sternberg PW (2001) Regulation of distinct muscle behaviors controls the *C. elegans* male’s copulatory spicules during mating. Cell 107: 777–788. 11747813 10.1016/s0092-8674(01)00600-6

[15] Chalfie M, Sulston JE, White JG, Southgate E, Thomson JN, et al (1985) The Neural Circuit for Touch Sensitivity in *Caenorhabditis elegans* . J Neurosci 5: 956–964. 3981252 10.1523/JNEUROSCI.05-04-00956.1985 PMC6565008

[16] Gray JM, Hill JJ, Bargmann CI (2005) A circuit for navigation in *Caenorhabditis elegans* . Proc Natl Acad Sci U S A 102: 3184–3191. 15689400 10.1073/pnas.0409009101 PMC546636

[17] Wicks SR, Roehrig CJ, Rankin CH (1996) A dynamic network simulation of the nematode tap withdrawal circuit: predictions concerning synaptic function using behavioral criteria. J Neurosci 16: 4017–4031. 8656295 10.1523/JNEUROSCI.16-12-04017.1996 PMC6578605

[18] Zheng Y, Brockie PJ, Mellem JE, Madsen DM, Maricq AV (1999) Neuronal control of locomotion in *C. elegans* is modified by a dominant mutation in the GLR-1 ionotropic glutamate receptor. Neuron 24: 347–361. 10571229 10.1016/s0896-6273(00)80849-1

[19] de Bono M, Maricq AV (2005) Neuronal substrates of complex behaviors in *C. elegans* . Annu Rev Neurosci 28: 451–501. 16022603 10.1146/annurev.neuro.27.070203.144259

[20] Haspel G, O'Donovan MJ, Hart AC (2010) Motoneurons Dedicated to Either Forward or Backward Locomotion in the Nematode *Caenorhabditis elegans* . J Neurosci 30: 11151–11156. 20720122 10.1523/JNEUROSCI.2244-10.2010 PMC2945236

[21] Kawano T, Po MD, Gao SB, Leung G, Ryu WS, et al (2011) An Imbalancing Act: Gap Junctions Reduce the Backward Motor Circuit Activity to Bias *C. elegans* for Forward Locomotion. Neuron 72: 572–586. 22099460 10.1016/j.neuron.2011.09.005

[22] Ben Arous J, Tanizawa Y, Rabinowitch I, Chatenay D, Schafer WR (2010) Automated imaging of neuronal activity in freely behaving *Caenorhabditis elegans* . J Neurosci Methods 187: 229–234. 20096306 10.1016/j.jneumeth.2010.01.011

[23] Chronis N, Zimmer M, Bargmann CI (2007) Microfluidics for in vivo imaging of neuronal and behavioral activity in *Caenorhabditis elegans* . Nat Methods 4: 727–731. 17704783 10.1038/nmeth1075

[24] Husson SJ, Costa WS, Wabnig S, Stirman JN, Watson JD, et al (2012) Optogenetic analysis of a nociceptor neuron and network reveals ion channels acting downstream of primary sensors. Curr Biol 22: 743–752. 22483941 10.1016/j.cub.2012.02.066 PMC3350619

[25] Piggott Beverly J, Liu J, Feng Z, Wescott Seth A, Xu XZS (2011) The Neural Circuits and Synaptic Mechanisms Underlying Motor Initiation in *C. elegans* . Cell 147: 922–933. 22078887 10.1016/j.cell.2011.08.053 PMC3233480

[26] Croll NA (1975) Components and patterns in the behaviour of the nematode *Caenorhabditis elegans* . J Zool 176: 159–176.

[27] Kleemann G, Basolo A (2007) Facultative decrease in mating resistance in hermaphroditic *Caenorhabditis elegans* with self-sperm depletion. Anim Behav 74: 1339–1347.

[28] Saifee O, Wei L, Nonet ML (1998) The *Caenorhabditis elegans unc-64* locus encodes a syntaxin that interacts genetically with synaptobrevin. Mol Biol Cell 9: 1235–1252. 9614171 10.1091/mbc.9.6.1235 PMC25346

[29] Nagel G, Brauner M, Liewald JF, Adeishvili N, Bamberg E, et al (2005) Light activation of channelrhodopsin-2 in excitable cells of *Caenorhabditis elegans* triggers rapid behavioral responses. Curr Biol 15: 2279–2284. 16360690 10.1016/j.cub.2005.11.032

[30] Nathoo AN, Moeller RA, Westlund BA, Hart AC (2001) Identification of neuropeptide-like protein gene families in *Caenorhabditis elegans* and other species. Proc Natl Acad Sci U S A 98: 14000–14005. 11717458 10.1073/pnas.241231298 PMC61156

[31] Edwards SL, Charlie NK, Milfort MC, Brown BS, Gravlin CN, et al (2008) A novel molecular solution for ultraviolet light detection in *Caenorhabditis elegans* . PLoS Biol 6: e198. 18687026 10.1371/journal.pbio.0060198 PMC2494560

[32] Zhang F, Wang LP, Brauner M, Liewald JF, Kay K, et al (2007) Multimodal fast optical interrogation of neural circuitry. Nature 446: 633–639. 17410168 10.1038/nature05744

[33] Zhao S, Cunha C, Zhang F, Liu Q, Gloss B, et al (2008) Improved expression of halorhodopsin for light-induced silencing of neuronal activity. Brain Cell Biol 36: 141–154. 18931914 10.1007/s11068-008-9034-7 PMC3057022

[34] Miller DM, Shen MM, Shamu CE, Burglin TR, Ruvkun G, et al (1992) *C. elegans unc-4* Gene Encodes a Homeodomain Protein That Determines the Pattern of Synaptic Input to Specific Motor Neurons. Nature 355: 841–845. 1347150 10.1038/355841a0

[35] Feng Z, Li W, Ward A, Piggott BJ, Larkspur ER, et al (2006) A *C. elegans* model of nicotine-dependent behavior: regulation by TRP-family channels. Cell 127: 621–633. 17081982 10.1016/j.cell.2006.09.035 PMC2859215

[36] Jones AK, Sattelle DB (2004) Functional genomics of the nicotinic acetylcholine receptor gene family of the nematode, *Caenorhabditis elegans* . Bioessays 26: 39–49. 14696039 10.1002/bies.10377

[37] Liu Y, LeBeouf B, Guo X, Correa PA, Gualberto DG, et al (2011) A cholinergic-regulated circuit coordinates the maintenance and bi-stable states of a sensory-motor behavior during *Caenorhabditis elegans* male copulation. PLoS Genet 7: e1001326. 21423722 10.1371/journal.pgen.1001326 PMC3053324

[38] Brockie PJ, Mellem JE, Hills T, Madsen DM, Maricq AV (2001) The *C. elegans* glutamate receptor subunit NMR-1 is required for slow NMDA-activated currents that regulate reversal frequency during locomotion. Neuron 31: 617–630. 11545720 10.1016/s0896-6273(01)00394-4

[39] Francis MM, Evans SP, Jensen M, Madsen DM, Mancuso J, et al (2005) The Ror receptor tyrosine kinase CAM-1 is required for ACR-16-mediated synaptic transmission at the *C. elegans* neuromuscular junction. Neuron 46: 581–594. 15944127 10.1016/j.neuron.2005.04.010

[40] Ballivet M, Alliod C, Bertrand S, Bertrand D (1996) Nicotinic acetylcholine receptors in the nematode *Caenorhabditis elegans* . J Mol Biol 258: 261–269. 8627624 10.1006/jmbi.1996.0248

[41] Touroutine D, Fox RM, Von Stetina SE, Burdina A, Miller DM, et al (2005) *acr-16* encodes an essential subunit of the levamisole-resistant nicotinic receptor at the *Caenorhabditis elegans* neuromuscular junction. J Biol Chem 280: 27013–27021. 15917232 10.1074/jbc.M502818200

[42] Barrios A, Ghosh R, Fang C, Emmons SW, Barr MM (2012) PDF-1 neuropeptide signaling modulates a neural circuit for mate-searching behavior in *C. elegans* . Nature Neurosci 15: 1675–1682. 23143519 10.1038/nn.3253 PMC3509246

[43] Chang AJ, Chronis N, Karow DS, Marletta MA, Bargmann CI (2006) A distributed chemosensory circuit for oxygen preference in *C. elegans* . PLoS Biol 4: e274. 16903785 10.1371/journal.pbio.0040274 PMC1540710

[44] Duerr JS, Han HP, Fields SD, Rand JB (2008) Identification of major classes of cholinergic neurons in the nematode *Caenorhabditis elegans* . J Comp Neurol 506: 398–408. 18041778 10.1002/cne.21551

[45] Brockie PJ, Maricq AV Ionotropic glutamate receptors: genetics, behavior and electrophysiology (January 19, 2006). *WormBook*, ed The *C elegans* Research Community, WormBook, doi/101895/wormbook11611, http://wwwwormbookorg (Accessed 2012 July 20). 10.1895/wormbook.1.61.1 PMC4781458 18050468

[46] Chalasani SH, Chronis N, Tsunozaki M, Gray JM, Ramot D, et al (2007) Dissecting a circuit for olfactory behaviour in *Caenorhabditis elegans* . Nature 450: 63–70. 17972877 10.1038/nature06292

[47] Ohnishi N, Kuhara A, Nakamura F, Okochi Y, Mori I (2011) Bidirectional regulation of thermotaxis by glutamate transmissions in *Caenorhabditis elegans* . EMBO J 30: 1376–1388. 21304490 10.1038/emboj.2011.13 PMC3094115

[48] Siehr MS, Koo PK, Sherlekar AL, Bian XL, Bunkers MR, et al (2011) Multiple doublesex-Related Genes Specify Critical Cell Fates in a *C. elegans* Male Neural Circuit. PLoS One 6: e26811. 22069471 10.1371/journal.pone.0026811 PMC3206049

[49] Whittaker AJ, Sternberg PW (2009) Coordination of opposing sex-specific and core muscle groups regulates male tail posture during *Caenorhabditis elegans* male mating behavior. BMC Biology 7: 33. 19545405 10.1186/1741-7007-7-33 PMC2715377

[50] Fayyazuddin A, Zaheer MA, Hiesinger PR, Bellen HJ (2006) The Nicotinic Acetylcholine Receptor Dα7 Is Required for an Escape Behavior in *Drosophila.* . PLoS Biol 4: e63. 16494528 10.1371/journal.pbio.0040063 PMC1382016

[51] Palikhova TA, Abramova MS, Pivovarov AS (2006) Cholinergic sensory inputs to command neurons in edible snail. Bull Exp Biol Med 142: 275–278. 17426828 10.1007/s10517-006-0345-3

[52] Yono O, Aonuma H (2008) Cholinergic neurotransmission from mechanosensory afferents to giant interneurons in the terminal abdominal ganglion of the cricket *Gryllus bimaculatus* . Zoolog Sci 25: 517–525. 18558805 10.2108/zsj.25.517

[53] Garrison JL, Macosko EZ, Bernstein S, Pokala N, Albrecht DR, et al (2012) Oxytocin/Vasopressin-Related Peptides Have an Ancient Role in Reproductive Behavior. Science 338: 540–543. 23112335 10.1126/science.1226201 PMC3597094

[54] Lee K, Portman DS (2007) Neural sex modifies the function of a *C. elegans* sensory circuit. Curr Biol 17: 1858–1863. 17964163 10.1016/j.cub.2007.10.015

[55] Liu Y, LeBoeuf B, Garcia LR (2007) G alpha(q)-coupled muscarinic acetylcholine receptors enhance nicotinic acetylcholine receptor signaling in *Caenorhabditis elegans* mating behavior. J Neurosci 27: 1411–1421. 17287516 10.1523/JNEUROSCI.4320-06.2007 PMC6673585

[56] Srinivasan J, Kaplan F, Ajredini R, Zachariah C, Alborn HT, et al (2008) A blend of small molecules regulates both mating and development in *Caenorhabditis elegans* . Nature 454: 1115–1118. 18650807 10.1038/nature07168 PMC2774729

[57] White JQ, Jorgensen EM (2012) Sensation in a Single Neuron Pair Represses Male Behavior in Hermaphrodites. Neuron 75: 593–600. 22920252 10.1016/j.neuron.2012.03.044 PMC3475325

[58] White JQ, Nicholas TJ, Gritton J, Truong L, Davidson ER, et al (2007) The sensory circuitry for sexual attraction in *C. elegans* males. Curr Biol 17: 1847–1857. 17964166 10.1016/j.cub.2007.09.011

[59] Hardaker LA, Singer E, Kerr R, Zhou GT, Schafer WR (2003) Serotonin modulates locomotory behavior and coordinates egg-laying and movement in *Caenorhabditis elegans.* . J Neurobiol 54: 537–537. 10.1002/neu.10014 11745666

[60] Clyne JD, Miesenböck G (2008) Sex-specific control and tuning of the pattern generator for courtship song in *Drosophila* . Cell 133: 354–363. 18423205 10.1016/j.cell.2008.01.050

[61] Pan Y, Meissner GW, Baker BS (2012) Joint control of *Drosophila* male courtship behavior by motion cues and activation of male-specific P1 neurons. Proc Natl Acad Sci U S A 109: 10065–10070. 22645338 10.1073/pnas.1207107109 PMC3382505

[62] von Philipsborn AC, Liu TX, Yu JY, Masser C, Bidaye SS, et al (2011) Neuronal Control of *Drosophila* Courtship Song. Neuron 69: 509–522. 21315261 10.1016/j.neuron.2011.01.011

[63] Lewis JA, Wu CH, Berg H, Levine JH (1980) The Genetics of Levamisole Resistance in the Nematode *Caenorhabditis elegans* . Genetics 95: 905–928. 7203008 10.1093/genetics/95.4.905 PMC1214276

[64] Lewis JA, Wu CH, Levine JH, Berg H (1980) Levamisole-Resistant Mutants of the Nematode *Caenorhabditis elegans* Appear to Lack Pharmacological Acetylcholine-Receptors. Neuroscience 5: 967–989. 7402460 10.1016/0306-4522(80)90180-3

[65] Granato M, Schnabel H, Schnabel R (1994) Genesis of an organ: Molecular analysis of the *pha-1* gene. Development 120: 3005–3017. 7607088 10.1242/dev.120.10.3005

[66] Hodgkin J, Horvitz HR, Brenner S (1979) Nondisjunction mutants of the nematode *Caenorhabditis elegans* . Genetics 91: 67–94. 17248881 10.1093/genetics/91.1.67 PMC1213932

[67] Mello CC, Fire A (1995) DNA Transformation. Methods Cell Biol 48: 451–482. 8531738

[68] Hobert O (2002) PCR fusion-based approach to create reporter gene constructs for expression analysis in transgenic *C. elegans* . Biotechniques 32: 728–730. 11962590 10.2144/02324bm01

[69] Bargmann CI, Avery L (1995) Laser killing of cells in *Caenorhabditis elegans.* . Methods Cell Biol 48: 225–250. 8531727 10.1016/s0091-679x(08)61390-4 PMC4442485

