# Supplementary figures and images for: The C. elegans Male Exercises Directional Control during Mating through Cholinergic Regulation of Sex-Shared Command Interneurons
Source: PLoS One. 2013 Apr 5;8(4):e60597. doi: 10.1371/journal.pone.0060597 (PMC3618225; doi:10.1371/journal.pone.0060597)

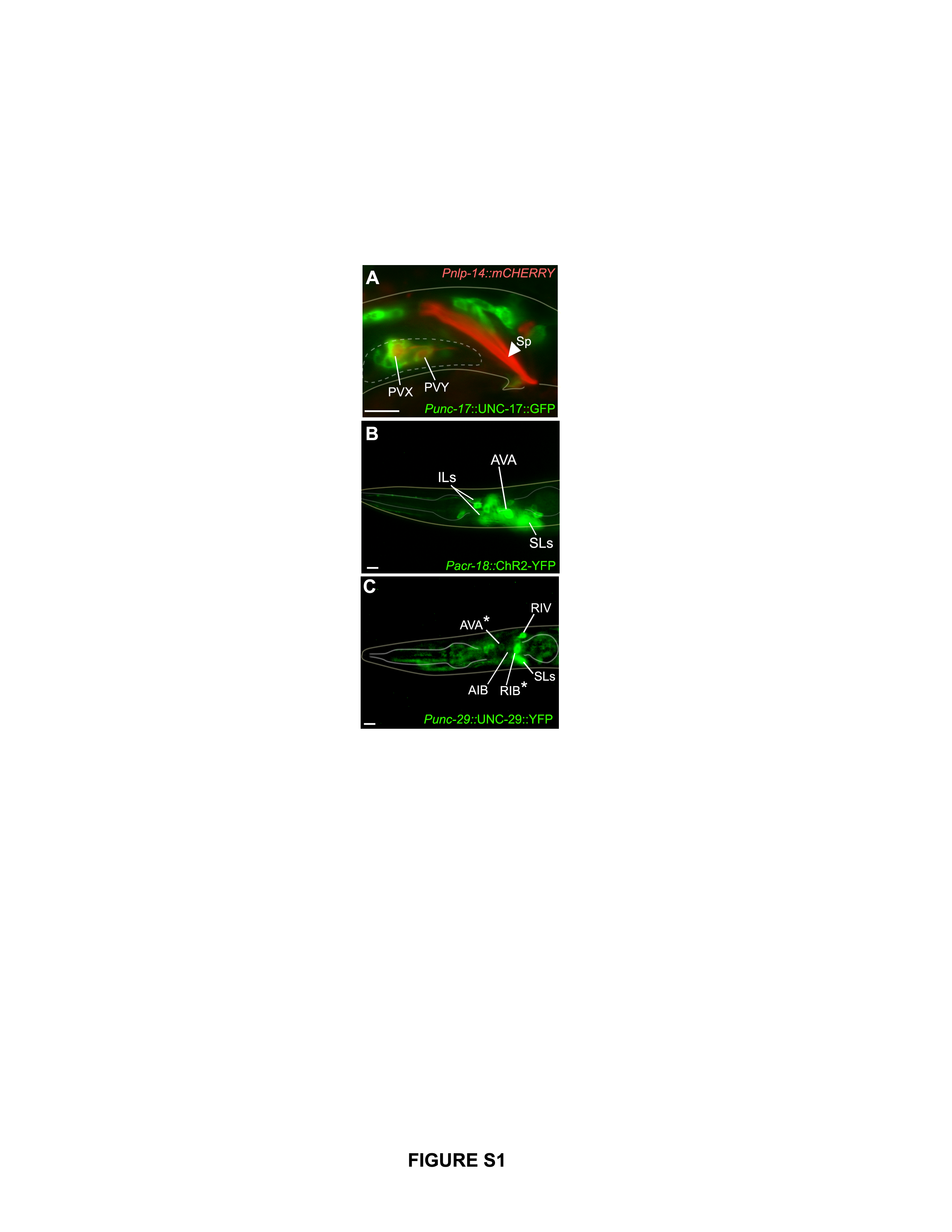

Supplement: Figure S1 — PVY and PVX are cholinergic and their command interneuron targets express cholinergic receptor genes. A. Fluorescent micrograph of an adult male tail showing co-expression of a Pnlp-14::mCHERRY reporter and a cholinergic marker (UNC-17::GFP) [14], [44] in PVY and PVX. The dotted line indicates the approximate boundary of the pre-anal ganglion (PAG) in which PVY and PVX reside. The spicules (SP) are visible due to their auto-fluorescent properties. B. Fluorescent micrograph of an L3 male showing expression of a Pacr-18::ChR2-YFP transgene in head neurons, including AVA. This reporter is also expressed in neurons of the ventral nerve cord (VNC) in both sexes and in a subset of ray and PAG neurons in the male (data not shown). C. Fluorescent micrograph of an L3 male showing expression of a full-length unc-29 translational reporter in neurons of the head. *, cells in which unc-29 and acr-16 reporters are co-expressed. The expression pattern of the unc-29 reporter in the hermaphrodite is superficially similar to that of the male. SLs in (B) and (C) correspond to sub-lateral neurons (possibly SMB/Ds, SIBs or SIAs). For all images the scale bar indicates 10 µm. (TIF) [file pone.0060597.s001.tif]

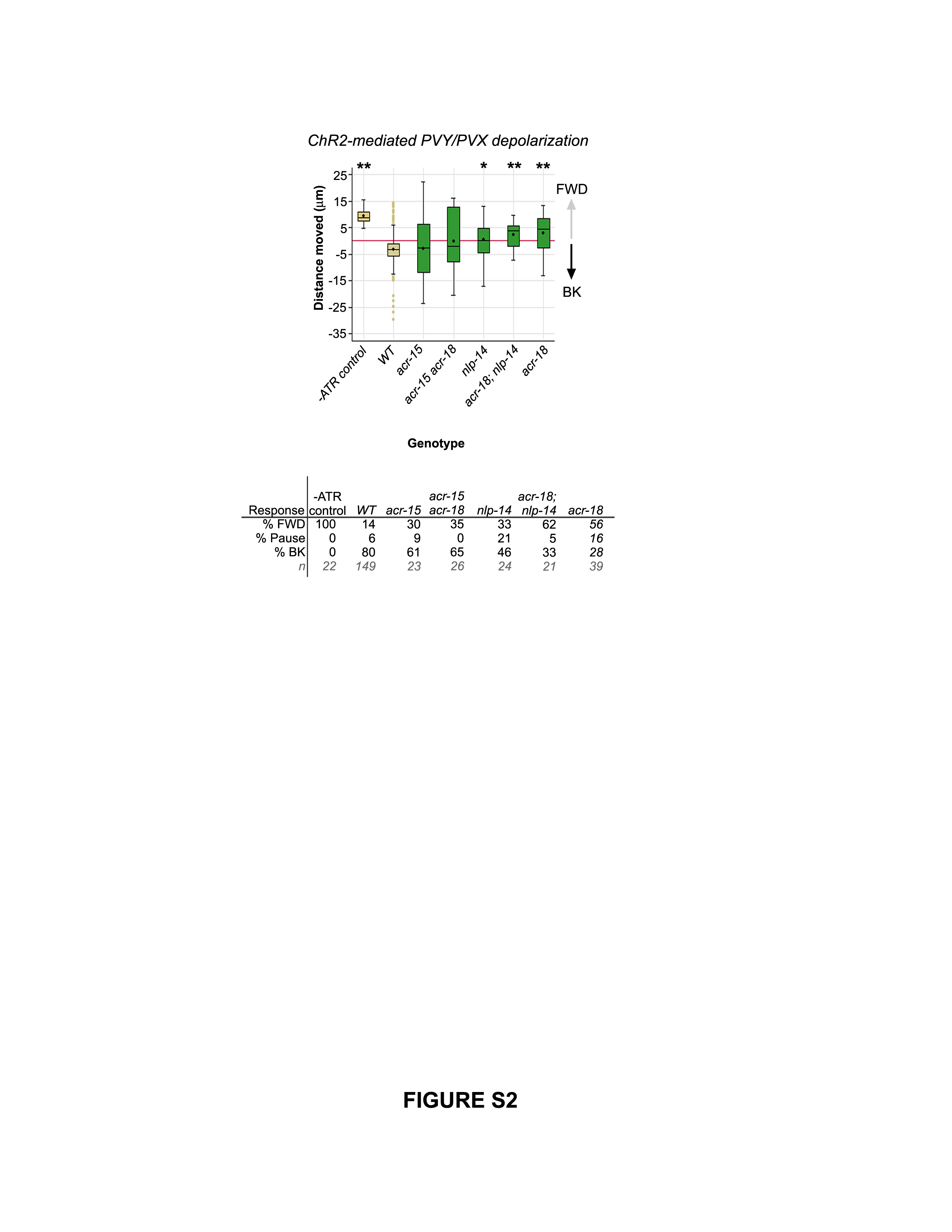

Supplement: Figure S2 — The impact of acr-15 and nlp-14 mutations on PVY+PVX-induced reversal behavior. Males of the genotype indicated on the X-axis and carrying the Pnlp-14::ChR2-YFP transgene were subjected to artificial activation assays. See legend of Fig. 3 for details of graph and table format and statistical analyses. Except for the “-ATR control”, all males were cultured and assayed in the presence of ATR. acr-15 has no significant impact on reversal behavior, either as a single mutation or in combination with acr-18 mutations, arguing that the absence of phenotype in acr-15 single mutants is not a consequence of functional redundancy with acr-18. In addition to cholinergic markers, PVY and PVX express the nlp(neuropeptide-like protein)-14 gene, which is predicted to encode neuropeptides with sequence similarity to orcokinin from Orconectes limosus (crayfish) [30]. nlp-14 mutants show impaired reversal response. acr-18; nlp-14 double mutants are phenotypically similar to acr-18 single mutants. This suggests that NLP-14 has a neuromodulatory role in PVY/PVX transmission and that cholinergic signaling (mediated by ACR-18- ACR-16- and UNC-29-containing receptors) is the rate-limiting factor. Treatments that were statistically different from wild type (WT) are indicated. Significance, *p<0.05; **p<0.005. (TIF) [file pone.0060597.s002.tif]
